# Digital Volume Correlation Challenge 2.0: A Comprehensive Dataset for Digital Volume Correlation Benchmarking

**DOI:** 10.21203/rs.3.rs-9683321/v1

**Published:** 2026-05-13

**Authors:** Zixiang Tong, Yujie Zhang, Edward Andò, Bin Chen, Brendan P. Croom, John O. Dabiri, Christian Franck, Matthew K. Fu, Helena Jin, Orion L. Kafka, Sriram Kunnoth, Thao D. Nguyen, Jacob Notbohm, Mohak Patel, Mainak Sarkar, Angkur J. Shaikeea, Jing Zhang, Alexander K. Landauer, Jin Yang

**Affiliations:** 1 Department of Aerospace Engineering and Engineering Mechanics, The University of Texas at Austin, USA; 2 EPFL Center for Imaging, École Polytechnique Fédérale de Lausanne (EPFL), Switzerland; 3 Wallenberg Wood Science Center, Department of Fiber and Polymer Technology, KTH Royal Institute of Technology, Sweden; 4 Research and Exploratory Development Department, Johns Hopkins Applied Physics Laboratory, USA; 5 Division of Engineering and Applied Science, California Institute of Technology, USA; 6 Department of Mechanical Engineering, University of Wisconsin-Madison, USA; 7 Sandia National Laboratory, USA; 8 Material Measurement Laboratory, National Institute of Standards and Technology, USA; 9 Currently: QuesTek Innovations, USA; 10 Department of Applied Mechanics, Indian Institute of Technology Delhi, India; 11 Division of Clinical Medicine, School of Medicine and Population Health, The University of Sheffield, UK; 12 Insigneo Institute, The University of Sheffield, UK; 13 Department of Mechanical Engineering, Johns Hopkins University, USA; 14 Google DeepMind, USA; 15 Carl R. Woese Institute for Genomic Biology, University of Illinois Urbana-Champaign, USA; 16 Texas Materials Institute, The University of Texas at Austin, USA; 17 The Wilmer Eye Institute, Johns Hopkins School of Medicine, USA

**Keywords:** Digital Volume Correlation, Benchmark dataset, Micro-X-ray computed tomography, Confocal microscopy, Synthetic image generation, Neutron imaging, Uncertainty quantification

## Abstract

**Background:**

Digital Volume Correlation (DVC) is a powerful experimental technique for quantifying 3D full-field volumetric displacements and strains. In light of its increased adoption in metrological applications, there is a critical need for benchmark datasets to systematically evaluate the performance of various DVC algorithms across different materials, imaging modalities, and deformation scenarios.

**Objective:**

Building on the foundations of DVC Challenge 1.0, the DVC Challenge 2.0 initiative aims to create a repository of DVC datasets to enable researchers to validate and refine their DVC algorithms against common benchmarks. This can help in expanding the scope and performance of DVC and foster innovation in volumetric deformation measurement.

**Methods:**

DVC Challenge 2.0 compiles a diverse collection of volumetric image sets contributed by the global research community. These datasets encompass different materials, loading conditions, and imaging modalities, including confocal/multiphoton microscopy, X-ray computed tomography (XCT), neutron tomography, and synthetically generated images. These datasets present various metrological challenges, such as complex deformation fields, poor image quality, and anisotropic or sparse speckle patterns. All datasets are published in an open repository, with a uniform image format, and a common data framework.

**Results:**

The resulting repository provides benchmark datasets for validating and comparing DVC algorithms, facilitating the exploration of DVC capabilities in diverse and challenging scenarios.

**Conclusion:**

By promoting collaboration and open data sharing, DVC Challenge 2.0 will drive innovation in volumetric deformation measurement techniques and broaden the impact of DVC. It will also help establish a baseline for comparison of DVC algorithms and codes.

## 1 Introduction

Digital Volume Correlation (DVC) is a experimental method for measuring three-dimensional, volumetric full-field deformations within solid materials [1-7]. Deformations are computed by tracking the motion of internal patterns or features within the contrast of 3D image volumes acquired at successive time points. These volumetric images are typically image stacks captured or reconstructed by X-ray computed tomography (XCT, including micro X-ray computed tomography or μCT) [1, 8-13], laser scanning confocal or multiphoton microscopy [14-19], magnetic resonance imaging (MRI) [20, 21], neutron tomography [22, 23], optical coherence tomography (OCT) [24-29] or other volumetric imaging techniques [6, 7, 30]. As a 3D extension of Digital Image Correlation (DIC) [31, 32], DVC offers significant advantages. Unlike 2D-DIC, which is limited to two in-plane deformations, or stereo-DIC that measures three components of displacement on a specimen surface [33], DVC quantifies the full 3D displacement internally, allowing all nine components of the strain tensor to be computed with sub-voxel resolution. While the mathematical formulation of DVC is derived from the same fundamental minimization principles as DIC [1, 3], its computational cost is substantially higher due to the added dimension. A key distinction lies in the diversity of image features encountered in DVC. For instance, confocal microscopy often utilizes fluorescent beads as fiducials to generate random 3D speckle patterns, analogous to paint dots used for DIC. Unlike DIC where random speckle patterns can usually be straightforwardly applied [34-38], many DVC applications are forced to rely on a material’s natural internal texture as the contrast agent, or fiducial, to track. These intrinsic patterns vary depending on the imaging instrument and the material being tested. Sparse or poorly conditioned feature patterns can lead to ill-posed optimization problems and introduce large measurement uncertainties. Therefore, there is a need for a set of comprehensive benchmarking datasets to include this wide spectrum of materials, imaging modalities, and deformation scenarios.

The first DVC Challenge (DVC Challenge 1.0) was organized to investigate measurement uncertainty and error sources across different laboratories, with a focus on XCT images [39]. Building upon the insights gained, we initiated the DVC Challenge 2.0 to systematically construct a public repository of representative and challenging DVC datasets to enable repeatable evaluation of DVC code performance. This initiative also has the potential to enable researchers worldwide to benchmark, validate, and refine their algorithms, fostering innovation and expanding the application of DVC in diverse experimental contexts. For example, this DVC Challenge 2.0 benchmarking dataset could further inspire the machine learning methods [40-42] to advance the post-processing accuracy and speed of DVC analysis, where training datasets can be generated or used based on real or realistic synthetic DVC images. The dataset repository is hosted by the National Institute of Standards and Technology (NIST) Public Data Repository and can be accessed via the link: https://data.nist.gov/od/id/mds2-4129.

This paper is structured as follows: Section 2 outlines the collection and analysis technique for the datasets, including the DVC algorithms employed and the metrics for quantifying measurement uncertainty and image features. Section 3 provides a detailed description of each dataset, including data source, materials, and collection methods, and is organized by imaging modality. It presents key characteristics and example results for each dataset. Section 4 presents a brief discussion of the noise analyses performed for the datasets. Finally, Section 5 discusses the implications of this benchmark collection and offers concluding remarks on future directions for the DVC community.

## 2 Methods

### 2.1 Collection of DVC Datasets

**Fig. 1:**
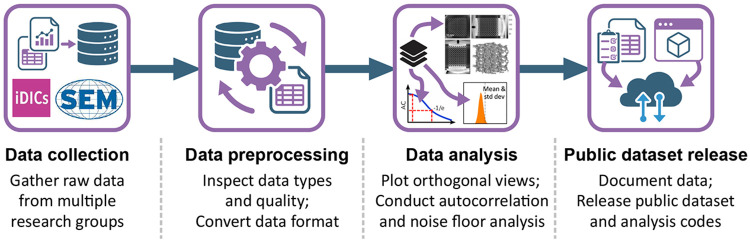
Workflow of the DVC Challenge 2.0 dataset pipeline. Volumetric datasets are first collected from multiple research groups, which were collected with a range of imaging modalities and materials. The data are then curated and preprocessed, including inspection of data types and quality control, followed by format standardization. In the analysis stage, orthogonal views are generated and quantitative image characterization is performed using autocorrelation and noise floor analysis. Finally, the curated datasets, documentation, and analysis codes are released as an open-access public repository to support benchmarking and development of DVC methods.

DVC Challenge 2.0 is part of the DIC Challenge Program (https://idics.org/challenge/) that is supported by the International Digital Image Correlation Society (iDICs) and the Society for Experimental Mechanics (SEM) [43-45]. For this DVC Challenge 2.0 benchmarking dataset topic, the organizers solicited participants to share DVC datasets at the iDICs and SEM annual conferences. An effort was made to cover multiple relevant types of imaging modalities. After agreeing to the terms of the collaboration, participants uploaded their datasets to a private file-sharing link hosted by the organizers. The organizers curated the datasets into a self-consistent, documented, freely available open-source resource [46], see Fig. 1 for an overview of this pipeline. The original images were use to make datasets consisting of converted images and analysis results, which were collated and organized by instrument type.

### 2.2 Analysis of DVC datasets

The format of all the collected datasets has been unified to use Tag Image File Format (TIFF or “.tif/.tiff”). TIFF files can be opened using open-source image viewing software such as ImageJ/FIJI [47], scientific computing packages like Python or MATLAB, and commercial DVC codes. We also provide MATLAB code to convert these TIFF files to the MATLAB “.mat” file format, and converted “.mat” versions of the images.

For a reproducible assessment of the collected datasets, we established a standardized processing, analysis, and reporting workflow. For each dataset, we first provide a summary table detailing specifications for the volumetric images, including material, imaging modality, image properties, and DVC image feature information, following the reporting recommendations from the iDICs DIC Good Practices Guide [31, 32]. To provide an intuitive visualization of each dataset, we present orthogonal views of a representative volume, typically the central slices along each axis, to show the internal details of the volume.

To provide a quantitative assessment, we evaluated the DVC measurement noise floor, as described in Section 2.3 “DVC Measurement Uncertainty Estimation”, and performed autocorrelation analysis to characterize the internal structure of the volumetric images, detailed in Section 2.4 “Estimating Image Features via Autocorrelation”. The DVC noise was computed using a two-step correlation procedure: an FFT-based cross correlation [14, 15, 48] was first used to obtain the initial displacement estimate, followed by an iterative Inverse Compositional Gauss-Newton (IC-GN) [3, 7, 49, 50] refinement to achieve sub-voxel accuracy. The autocorrelation was computed in Fourier space and averaged radially in real space. For clarity, spatial quantities expressed in voxel units are denoted as “vx” (voxel units) throughout this work, to distinguish them from physical units (e.g., μm).

### 2.3 DVC Measurement Uncertainty Estimation

For DVC measurements, similarly to DIC measurements, quantifying the measurement uncertainty under idealized static conditions is important, as it reflects the quality of the imaging system and the best-case reliability of the DVC results [51, 52]. Ideally, static measurements should yield zero displacements in the absence of applied motion. However, in practical experiments, non-zero displacements may arise due to image noise, system drift, thermal strains, or other measurement artifacts [39]. Lengthy imaging times and expensive imaging runs also limit the practicality of obtained static reference images.

To evaluate DVC measurement uncertainty from a nominally zero-motion (i.e., static) or rigid-body known displacement image pair, we quantify two key metrics: *systematic bias* (i.e., mean error) and *noise floor* (i.e., standard deviation). The systematic bias, $\mu ={\left[\mu_{x},\mu_{y},\mu_{z}\right]}^{T}$, defined as the mean error of the measured displacements, represents non-random deviations that may arise from, for example, optical distortions, system drift, or calibration imperfections. The noise floor, $\sigma ={\left[\sigma_{x},\sigma_{y},\sigma_{z}\right]}^{T}$, is characterized by the standard deviation of the displacements, representing the level of random noise in the measurement system. These metrics are computed from the displacements $u={\left[u_{x},\;u_{y},\;u_{z}\right]}^{T}$ between two nominally identical or rigid-body motion only images and are defined as:

(1) $$\mu =\frac{1}{N}\sum_{i=1}^{N}\left(u_{\text{DVC}}^{\left(i\right)}-u_{\text{Nominal}}^{\left(i\right)}\right)$$

where $u_{\text{DVC}}^{\left(i\right)}$ represents the displacement computed by the DVC method at point i on the DVC mesh; $u_{\text{Nominal}}^{\left(i\right)}$ is a ground truth displacement obtained from, for example, a translation stage with displacement accuracy five or more times that of the accuracy need for the DVC measurement. Often, this can be straightforwardly accomplished by a built-in specimen stage for XCT or microscope instruments. For nominally zero motion noise floor images u is taken to be identically zero. N is the total number of measurement points in the DVC region of interest (ROI).

In this work the noise floor is defined as the standard deviation of the DVC error after removing rigid body translation ^1^ $\sigma =\{\sigma_{j}\}\;(j=x,y,z)$, which carries information on the non-uniformity or noise in the measured data, given by:

(2) $$\sigma_{j}=\sqrt{\frac{1}{N}\sum_{i=1}^{N}{|u_{j\;\text{DVC}}^{\left(i\right)}-u_{j\;\text{Nominal}}^{\left(i\right)}-\mu_{j}|}^{2}}.$$

where $\mu_{j}$ denotes the j-th component of μ defined in Eq. (1).

If a dataset includes at least two static volumetric images, we use them directly for this assessment. For datasets without repeated static images, we generate synthetic static images by adding different levels of white Gaussian noise (1 %, 3 %, 5 %, and 10 % levels) to the original reference image. Each voxel is then rounded to an integer grayscale intensity before conducting noise floor analysis. The added Gaussian noise is scaled based on the observed intensity range of the reference image rather than the full bit-depth range to ensure a consistent signal-to-noise ratio. Specifically, for an image with an intensity range of [$I_{\text{min}}$, $I_{\max}$], the noise standard deviation $\sigma_{noise}$ is set as a fraction of this range: where k is chosen from [1, 3, 5, 10] % noise levels. Note that when Gaussian noise is added, only the noise floor can be estimated, as the noise does not introduce systematic bias. Therefore, systematic bias is marked as not applicable (N/A) in those tables. Also, importantly, while this method of adding synthetic noise is unlikely to accurately capture the experimental noise for a given image set, it is included to help the user understand what the sensitivity to noise is for a given combination of image, pattern, algorithm, and preprocessing types.


(3) $$\sigma_{noise}=k\times (I_{\max}-I_{\text{min}}),$$

**Fig. 2:**
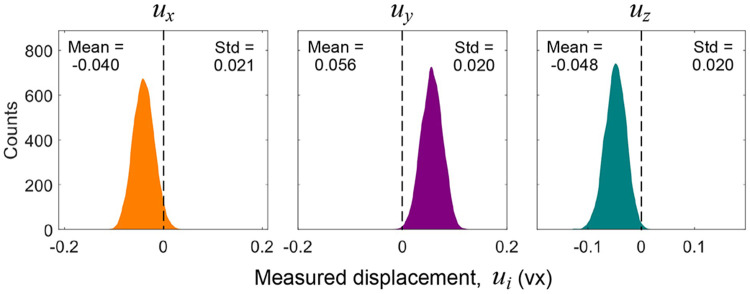
Statistical distributions of measured displacements from static DVC analysis using the DVC1 S4 dataset from DVC Challenge 1.0 [39]. The histograms show the distribution of displacement components measured between two nominally identical reference images. The systematic bias (mean values) and noise floor (standard deviation) are indicated for each displacement component.

As an example case the DVC Challenge 1.0 XCT Set 4 (DVC1 S4) dataset [39] is used as an example to illustrate noise analysis. Figure 2 shows the statistical distribution of displacement for a nominally zero motion experiment, where the horizontal axis represents the DVC measured displacement, and the vertical axis shows the binned counts of points in the DVC measurement ROI. The mean of the distribution indicates systematic bias, while the standard deviation indicates the measurement noise floor. Instead of displaying the statistical distribution of displacement for all datasets, the present the systematic bias and noise floor is reported as two rows of the descriptive table of each subsection.

### 2.4 Estimating Image Features via Autocorrelation

To estimate the intrinsic characteristics of 3D image features, such as their size, periodicity, and isotropy, we performed a 3D autocorrelation analysis following methods established in prior works on image-based structural quantification [34, 53-56]. The autocorrelation function describes how rapidly grayscale intensity values decorrelate as spatial separation increases [57, 58]. In structured volumetric images, nearby voxels (vx) belonging to the same microstructural feature tend to exhibit similar intensities, resulting in high autocorrelation values. As the separation distance exceeds the characteristic feature size, voxel intensities become increasingly statistically independent, and the autocorrelation decays toward zero. The rate of this decay, therefore, provides a measure of characteristic feature size and overall structural organization.

Accordingly, autocorrelation lengths defined at selected autocorrelation function thresholds serve as natural descriptors of structural texture and characteristic length scales. Larger autocorrelation lengths indicate coarser, more spatially persistent features, whereas shorter lengths correspond to finer, more rapidly varying textures. An intuitive analogy can be drawn from a forest landscape: at small offsets, autocorrelation length reflects correlations within a single tree and captures intra-feature structure. As r increases, the correlation increasingly reflects the spacing between neighboring trees, analogous to mesoscale organization in the image texture, such as clustered pores or structural domains. In this work, we establish three autocorrelation thresholds, 1/e (≈ 0.368), 0.10, and 0.01, which respectively characterize feature-scale, mesoscale, and far-tail correlation regimes. Note that the thresholds define three operational correlation regimes rather than physically distinct transitions or features.

Mathematically, for a given separation distance r and a polar angle θ, the autocorrelation function, AC(r,θ), quantifies the normalized similarity between voxel intensities separated by that distance and direction [53]:

(4) $$AC(r,\theta )=\frac{{ℱ}^{-1}\left\{{|ℱ[I(x)]|}^{2}\right\}(r,\theta )}{{ℱ}^{-1}\left\{{|ℱ[I(x)]|}^{2}\right\}(0,\theta )},$$

where I(x) denotes the grayscale intensity of the image at voxel position x; ℱ and ${ℱ}^{-1}$ represent the Fourier and inverse Fourier transforms. This formulation leverages the convolution theorem to efficiently compute voxel-wise spatial correlations via fast Fourier transforms (FFT). Additional implementation details are provided in Supplementary Material Section S1.

**Fig. 3:**
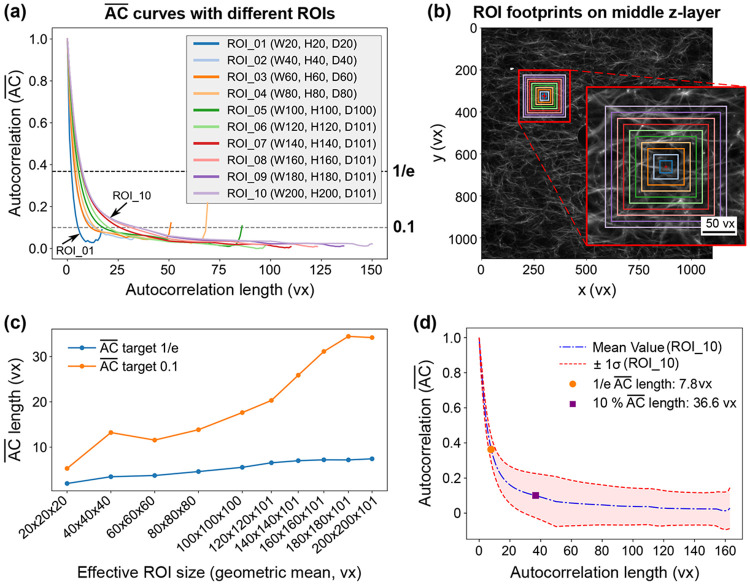
Multi-scale autocorrelation analysis and representative volume convergence. (a) Autocorrelation ($\bar{AC}$) curves computed for progressively increasing concentric ROI sizes. Horizontal dashed lines indicate the 1/e and 0.1 correlation thresholds used to define feature-scale and mesoscale characteristic lengths, respectively. (b) Spatial footprints of the concentric ROIs overlaid on the mid–*z* slice of the 3D volume, illustrating incremental expansion of the analyzed region. (c) Convergence of autocorrelation lengths at the 1/e and 0.1 thresholds as a function of effective ROI size (geometric mean dimension). Stabilization of these lengths identifies the feature-scale and mesoscale representative volume elements (RVEs). (d) Angularly averaged radial autocorrelation curve for the converged ROI (“ROI 10” in (a)) with a ±1 standard deviation (σ) envelope. The extracted 1/e and 0.1 correlation lengths are indicated, quantifying characteristic feature size and mesoscale organization.

For the analyses presented in this work, the autocorrelation function was angularly averaged over all directions to obtain a radial autocorrelation function

(5) $$\bar{AC}(r)=\frac{\int AC(r,\theta )\;d\theta }{\int d\theta },$$

which describes the mean decay of voxel similarity as a function of spatial separation. In Fig. 3(d), the plotted mean curve represents the angularly averaged radial autocorrelation function $\bar{AC}(r)$. The ±1 standard deviation envelope quantifies the variability of the autocorrelation values across angular directions prior to averaging.

To preserve spatial locality and avoid excessive global averaging, each volumetric dataset was partitioned into concentric ROIs (see Fig. 3(b)). Each ROI expands incrementally with a radius step Δr, enabling systematic evaluation of how spatial correlation statistics evolve with increasing observation volume. The resulting ROI-specific autocorrelation curves ($\bar{AC}$ vs. r) characterize the decay of voxel similarity with distance, from which multiple quantitative descriptors of feature and mesoscale structure are extracted (see Table 1).

#### 2.4.1 Feature-scale RVE and characteristic feature size

The primary decay in each autocorrelation function curve corresponds to the “$\bar{AC}(r)=1∕e$” level, a typical definition of correlation length in spatial statistics. At this separation distance, voxel intensity correlations decay to approximately one-third of their initial value, indicating that spatial similarity beyond individual features has significantly diminished. The corresponding offset defines the characteristic feature size.

**Fig. 4:**
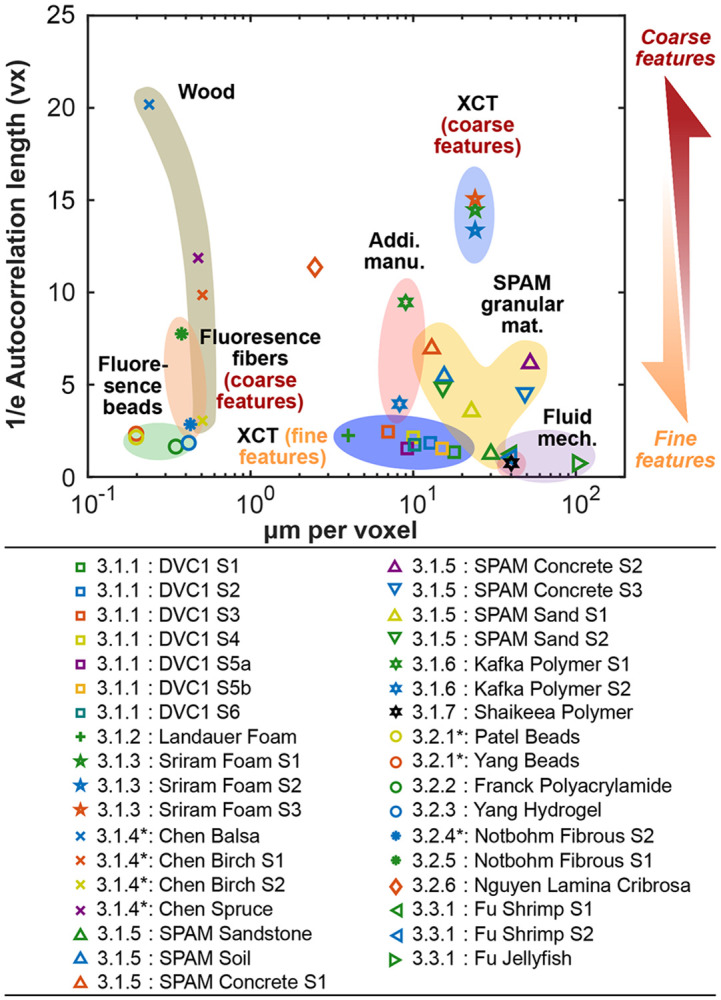
The overview map of the DVC Challenge 2.0 dataset images, plotted according to their physical resolution (μm per voxel) and characteristic feature size (1/e autocorrelation length). A larger 1/e autocorrelation length corresponds to larger or rougher image features, which typically present a greater challenge for DVC algorithms, while a smaller value indicates finer, smaller features. Each symbol represents a specific dataset as detailed in the legend, and the shaded regions group datasets by material type or imaging modality. Legend entries denoted with a “*” symbol indicate computationally generated (synthetic) data.

As shown in Fig. 3(c), the 1/e crossing length initially varies with ROI size but converges as the ROI becomes sufficiently large. The smallest ROI at which this correlation length stabilizes defines the feature-scale representative volume element (RVE), the minimum volume required to obtain statistically representative feature-scale texture measurements. These characteristic lengths are further incorporated into the dataset overview map (Fig. 4) to relate image texture statistics to dataset resolution and material structure.

#### 2.4.2 Mesoscale RVE and mesoscale structural length

Mesoscale organization, including clustered pores, layered structures, or network connectivity, is characterized using the “$\bar{AC}(r)$ = 0.10” threshold, which captures longer-range structural correlations beyond individual features. The 0.10 threshold was selected as a practical long-range correlation cutoff that remains sufficiently above the noise-dominated regime while capturing mesoscale structural organization. As illustrated in Fig. 3(c), the corresponding correlation length typically exceeds the feature-scale value and reflects mesoscale spatial organization.

The smallest ROI at which the 0.10 correlation length stabilizes defines the mesoscale RVE, while the associated offset provides the mesoscale structural length scale. In the forest analogy, this corresponds to the characteristic spacing between clusters of trees or clearings. If convergence is not observed within the available field of view, the mesoscale length is reported with the caveat that mesoscale statistics remain dependent on observation volume.

#### 2.4.3 Far-tail behavior and data quality diagnostics

The far-tail regime, defined by the “$\bar{AC}(r)$ = 0.01” threshold, serves primarily as a diagnostic indicator of data quality and image drift. Ideally, the autocorrelation function approaches zero at large separations. Persistent nonzero values may indicate illumination gradients, imaging drift, or finite-volume truncation effects. Because this regime is highly sensitive to noise, it is used only as a qualitative diagnostic rather than a quantitative descriptor of structural length scales.

#### 2.4.4 Directional dependence and quasi-periodic correlation behavior

Beyond scalar correlation length scales, the full autocorrelation function also contains information about the quasi-periodicity and directional dependence of the material’s microstructures: (i) *Quasi-periodic* structural organization is inferred from oscillatory features in the autocorrelation function, where recurrent peaks and troughs indicate characteristic inter-feature spacing or repeated structural motifs. (ii) *Directional dependence* of spatial correlation was examined by computing autocorrelation functions along multiple angular directions. The mean autocorrelation curve and its ±1 standard deviation envelope summarize the variability of correlation behavior across directions. However, directional variability alone does not necessarily indicate anisotropy, as random texture variations may produce similar dispersion. Anisotropy is inferred only when consistent directional differences are observed in the correlation decay, or in the extracted correlation lengths, indicating preferential structural alignment.

Figure 3(d) illustrates these descriptors for a representative converged ROI, showing the mean autocorrelation function together with its ±1 standard deviation envelope, as well as the identified 1/e and 0.10 correlation lengths. For conciseness, the main summary tables in this paper report only the 1/e and 0.10 correlation lengths in the respective tables.

This multi-scale autocorrelation framework forms the foundation of our structural characterization methodology. By systematically analyzing spatial correlations across increasing ROI sizes, we (i) distinguish feature-scale and mesoscale structure, (ii) identify representative volume elements, (iii) diagnose long-range imaging artifacts, and (iv) qualitatively characterize quasi-periodic organization and directional dependence (i.e., feature anisotropy). This approach ensures that all reported structural metrics are statistically representative, physically meaningful, and computationally robust.

### 2.5 FFT-based and IC-GN-based DVC Algorithms

Most DVC methods are broadly categorized as local [1, 3, 59], global [4, 60, 61], or hybrid methods [50, 62]. Local-DVC methods are the most widely used and serve as the standard approach for post-processing in this paper ^2^ . In local DVC methods, the volume is divided into smaller subvolumes (subsets), and the displacement vector is solved independently for each subset. A straightforward, fast implementation uses FFT-based cross-correlation [14, 62, 65], where the peak of the cross-correlation matrix indicates the best match. The cross-correlation function can be written using Fourier transforms as follows

(6) $$C_{\text{CC}}(u)={ℱ}^{-1}\left[\bar{ℱ(f)}⊙ℱ(g)\right],$$

where f and g represent the grayscale values of the reference and deformed volume images, respectively, $\left(\bar{\cdot }\right)$ denotes the complex conjugate, and (⊙) is the Hadamard product, where multiplication is conducted element-wise. ℱ(•) and ${ℱ}^{-1}(•)$ represent the Fourier transform and its inverse. The rigid translation vector u is estimated from the location of the cross-correlation peak relative to the origin. In practice, another modified version of Eq (6) called zero-mean normalized cross correlation (ZNCC) is also widely used as the most robust but more computationally expensive option [66]:

(7) $$C_{\text{ZNCC}}=\frac{1}{\sigma_{f}\sigma_{g}}\sum_{i=-M}^{M}\sum_{j=-M}^{M}\sum_{k=-M}^{M}\left[f(x_{0}+i,\;y_{0}+j,\;z_{0}+k)-f_{m}\right]\left[g(x_{0}^{\prime }+i,\;y_{0}^{\prime }+j,\;z_{0}^{\prime }+k)-g_{m}\right],$$

where f(x,y,z) is the gray level intensity at coordinates (x,y,z) in the subset of the reference image, and $g(x^{\prime },y^{\prime },z^{\prime })$ is the gray level intensity at coordinates ($x^{\prime }$, $y^{\prime }$, $z^{\prime }$) in the target subset of the deformed image. The calculation is performed over a cubic subset of size (2M+1)×(2M+1)×(2M+1)vx, where M is an integer defining the half-width of the subset. $f_{m}$ and $g_{m}$ are the mean intensity values of reference and target subsets, respectively, that are defined as

(8) {fm=1(2M+1)3∑i=−MM∑j=−MM∑k=−MMf(x0+i,y0+j,z0+k)gm=1(2M+1)3∑i=−MM∑j=−MM∑k=−MMg(x0′+i,y0′+j,z0′+k).}

Symbols $\sigma_{f}$ and $\sigma_{g}$ are variances of the local reference subset and deformed subset grayscale values. These, in turn, are defined as

(9) {σf=∑i=−MM∑j=−MM∑k=−MM[f(x0+i,y0+j,z0+k)]2σg=∑i=−MM∑j=−MM∑k=−MM[g(x0′+i,y0′+j,z0′+k)]2.}

FFT-based DVC is computationally efficient. However, it is limited to cases with small translations and rotations, unless an iterative framework is adopted, since finite deformations lead to decorrelation and inaccurate matches [62, 67]. An alternative is the inverse compositional Gauss-Newton (IC-GN) algorithm [49, 68], which uses higher-order shape functions to approximate the local displacement field. A cost function, such as the zero-normalized sum of squared differences (ZNSSD) [3, 49], is minimized to find the optimal match between the reference and deformed subsets, which is defined as

(10) $$C_{\text{ZNSSD}}=\sum_{i=-M}^{M}\sum_{j=-M}^{M}\sum_{k=-M}^{M}{\left[\frac{f(x_{0}+i,\;y_{0}+j,\;z_{0}+k)-f_{m}}{\sigma_{f}}-\frac{g(x_{0}^{\prime }+i,\;y_{0}^{\prime }+j,\;z_{0}^{\prime }+k)-g_{m}}{\sigma_{g}}\right]}^{2}.$$

where coordinates $X={\left[x_{0},y_{0},z_{0}\right]}^{T}$ and $y(X)={\left[x_{0}^{\prime },y_{0}^{\prime },z_{0}^{\prime }\right]}^{T}$ represent the centroid of a reference subset and the corresponding deformed volume. Thus, y(•) represents the mapping from the reference coordinates to the deformed coordinates of any voxel. Solving the DVC problem is equivalent to minimizing the cost function to find the ideal mapping y [50]. Taking a first-order affine shape function as an example, y can be expressed as

(11) $$y(X)=X+u(X)=X+\sum_{i=1}^{N}\chi (X)\;(u_{i}+F_{i}\;(X-X_{i0})),$$

where χ(X) equals 1 if a point X is within the subset i; $X_{i0}$ denotes the center point of the subset i; $u_{i}$ is the displacement of $X_{i0}$ in the $e_{i}$ direction, and $F_{i}$ represents the affine displacement gradient tensor minus the identity tensor. Together, $u_{i}$ and $F_{i}$ include 12 degrees of freedom in total: $p={\left[u_{x},\;u_{y},\;u_{z},\;F_{xx},\;F_{xy},\;F_{xz},\;F_{yx},\;F_{yy},\;F_{yz},\;F_{zx},\;F_{zy},\;F_{zz}\right]}^{T}$. The problem then reduces to minimizing the ZNSSD cost function to determine these 12 coefficients optimally. The IC-GN algorithm is widely used to solve this optimization problem efficiently [50, 68-75].

## 3 Description of Datasets

Figure 4 provides an overview of the entire benchmark collection, organizing the diverse datasets based on two fundamental properties: the physical resolution of the imaging system and the nature of the internal features (i.e., material microstructures). The horizontal axis shows that the datasets span several orders of magnitude in spatial resolution, from the sub-micron voxel sizes typical of fluorescence microscopy to resolutions exceeding 100 μm per voxel for large-scale scans of granular materials and fluid mechanics experiments. This range ensures that the collection is relevant for applications over a wide range of experimental scales. The vertical axis differentiates the datasets based on their internal texture or fiducial, as quantified by the 1/e (≈ 0.368) autocorrelation length. Datasets with dense features, such as those from confocal microscopy of fluorescent beads or XCT scans of fine-grained materials, are clustered at the bottom. These images provide rich, well-distributed information for correlation. In contrast, datasets with sparse features, like reticulated open-cell foams, wood microstructure, and certain granular materials, occupy the upper region of the plot. These are inherently more difficult to analyze with DVC, often requiring larger subset sizes or more advanced algorithms to overcome the lack of texture. Each dataset is described in detail in this section, each with figures showing views of an example image, the autocorrelation analysis, and a table of the image, imaging, and example DVC analysis parameters.

### 3.1 Micro X-ray and Neutron Computed Tomography

#### 3.1.1 Syntactic foam rigid body motion (DVC Challenge 1.0 [39])

This dataset originates from the DVC Challenge 1.0, which is an interlaboratory study to evaluate DVC accuracy using XCT scans of lightweight syntactic foam [39]. The dataset has been publicly released and was originally reported by Croom et al. [76]. The specimens were made by embedding hollow glass microballoons (GMBs) in an elastomer matrix, creating a high-contrast three-dimensional microstructure that provides a strong natural speckle pattern. The dataset includes XCT scans acquired using seven different XCT systems across six participating laboratories. The imaging experiments include repeat scans (no rigid body motion), axial translations, and radial translations. Note that axial translation refers to the movement of the sample along the scanner’s axis of rotation, whereas radial translation refers to the movement of the sample in a direction orthogonal (perpendicular) to the axis of rotation.

**Fig. 5:**
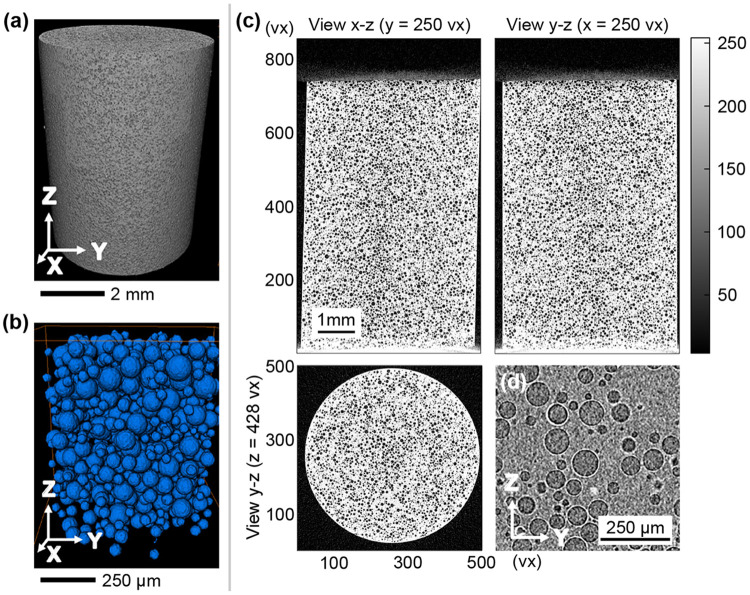
This figure presents various X-ray computed tomography (XCT) scans of the syntactic foam specimen. (a) An overall 3D rendering of DVC1 S3 at a 7.03 μm voxel resolution. (b) A high-resolution (1.7 μm/voxel) 3D view of the embedded hollow glass microballoons (GMBs). (c) Orthogonal views of the reference volumetric image from the DVC Challenge 1.0 Dataset XCT 4 (DVC1 S4) [39]. The horizontal and vertical axes denote spatial coordinates in voxel (vx) units. The colorbar represents grayscale intensity values of the volumetric image. (d) A high-resolution XCT cross-section (1.7 μm/voxel) of the syntactic foam, showing rhe embedded hollow glass microballoons (GMBs)[39]. (Images are adapted from Croom, B.P. et al. [39].)

A 3D rendering of the syntactic foam specimen obtained from XCT imaging is shown in Fig. 5(b). The cylindrical samples had a diameter of 4.8 mm and a height of 7 mm. The internal microstructure contained approximately 37 % volume fraction of hollow glass microballoons with diameters ranging from 30 μm to 95 μm. The strong contrast between the hollow GMBs and the surrounding elastomer matrix produces a high-quality three-dimensional texture that is well-suited for displacement tracking using DVC.

The reconstructed voxel unit sizes range from 7 μm to 18 μm, depending on the system. For the data discussed here, Dataset DVC1 S4 is selected as a representative example. Three orthogonal slices of the S4 reference volume are shown in Fig. 5(c). Imaging and analysis parameters for all seven XCT systems participating in the DVC Challenge 1.0 are summarized in Table 2. The table reports the volumetric image dimensions, voxel resolutions, and characteristic autocorrelation length scales for each dataset, along with representative DVC processing parameters and resulting displacement bias and noise levels. As shown in the table, the autocorrelation analysis of the S4 reference volume yields characteristic lengths of approximately 2.2 vx at the 1/e threshold and 4.2 vx at the 10 % threshold (see Fig. S2 of the Supplementary Material).

This dataset provides a unique set of well-controlled and DVC-specific images that are expressly designed to understand the noise floor associated with XCT measurements. The images in this collection can readily be used to test or compare DVC codes or algorithms as a baseline.

#### 3.1.2 Elastomeric polymer foam under uniaxial compression (Landauer et al. [12, 77])

This dataset consists of a series of XCT images of an elastomeric polymer foam undergoing uniaxial stress compression that were collected and released in a foam materials dataset and data framework [12, 77]. This is an example of a nominally homogeneous macroscopic strain field applied to an inhomogeneous yet isotropic material. In this case, the intrinsic microstructural features of the foam act as the pattern used for DVC tracking. It contains seven images at nominal strains of [0.0, 7.0, 14, 21, 28, 35, 60] %. The displacement uncertainty (actuator resolution) is 3 μm, and the stage linearity is 0.1 % of the full scale of the 10 mm range. After reconstruction, images were combined from a .tif image sequence into an image stack. Images were normalized using the built-in ImageJ tool “Enhance Contrast” to have 0.10 % saturated pixels and an equalized histogram over the entire stack. The primary data collection was conducted by Orion Kafka and Newell Moser at NIST.

**Fig. 6:**
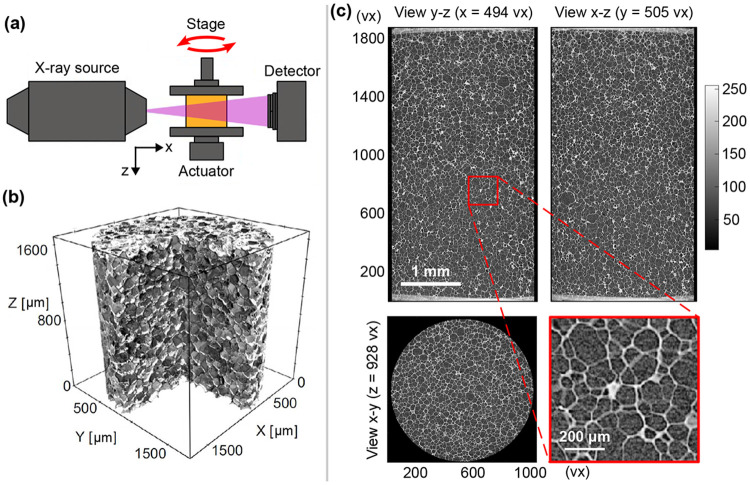
Overview of the closed-cell elastomeric foam experiment. (a) Illustrations of experimental setup of uniaxial compression test of elastomeric polymer foam XCT experiments. (b) 3D rendering of the reference XCT images (Images are adapted from Landauer et al. [12].) (c) Orthogonal views of the reference volumetric image for [12]. The red square region in the x-z view is zoomed in on in the right bottom subfigure. The horizontal and vertical axes denote spatial coordinates in voxel (vx) units. The colorbar represents grayscale intensity values of the volumetric image.

The key image parameters are given in Table 3, with more details and rendered views provided in the dataset notes and in [77]. Figure 6 illustrates the experimental setup and representative XCT images of the elastomeric foam specimen. A selected region in the x–z view is highlighted to illustrate the internal foam microstructure in greater detail.

**Fig. 7:**
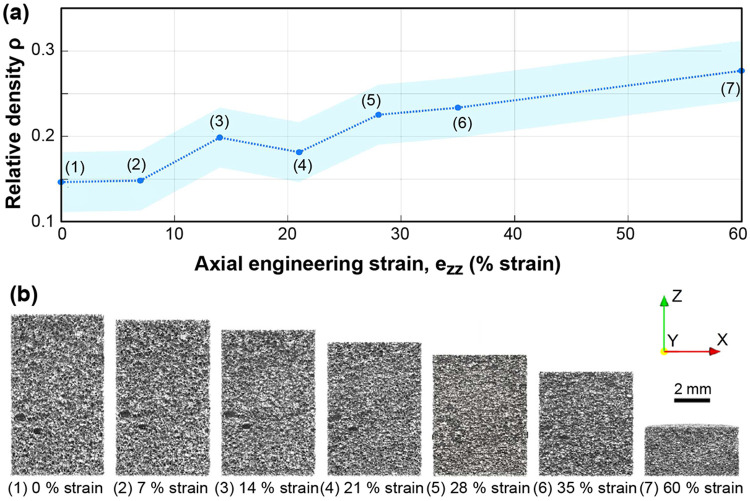
Summary of the deformation applied to images in the dataset. (a) Applied deformations from image 0 (undeformed reference) to image 7 (60 % nominal strain) in [77]. (b) The images show vertical slices of the reconstructed volume image of the foam at each strain step. These images show the center plane of each specimen. They can be used to, for example, identify morphology and deformation habits of specific features within the 3D structure or investigate inhomogeneities (e.g., compaction banding) during the deformation. (Figures are adapted from [12].)

The 1/e, 10 %, and 1 % $\bar{AC}$ lengths of the reference volume are 2.3 vx, 5.0 vx, and 12.5 vx, respectively (see the Supplementary Material Fig. S3 for the full autocorrelation curve). The applied averaged axial engineering strain from DVC results from image 0 (undeformed reference) to image 7 (60 % nominal strain) in [77] is plotted in Fig. 7(a). Figure 7(b) visualizes vertical slices of the reconstructed volume image of the foam at each strain step. These images show the center plane of each specimen. They can be used to, for example, identify morphology and deformation habits of specific features within the 3D structure or investigate inhomogeneities (e.g., compaction banding) during the deformation.

The key imaging and analysis parameters of the dataset are summarized in Table 3. The elastomeric foam images were acquired using XCT with a voxel size of 4 μm in all spatial directions. The volumetric stacks contain approximately 987 vx × 1009 vx × 1856 vx. The feature-scale correlation length (defined using the 1/e autocorrelation threshold) is approximately 2.3 vx, while the mesoscale correlation length (defined using the 10 % threshold) is approximately 5.0 vx.

The displacement noise floor estimated from the DVC analysis is small, on the order of 0.001 vx or lower in all three spatial directions. This dataset, therefore, provides a useful low-noise benchmark for evaluating DVC performance in materials with inherent contrast patterns that exhibit complex microstructural deformation but maintain a nominally uniform macroscopic strain field. It also provides a finite deformation test case with large compressive strains up to about 60 % compressive engineering strain.

#### 3.1.3 Open-cell foam multi-step compression experiment (Sriram et al. [78])

This dataset is an XCT volumetric image series of open-cell foam from a multi-step compression experiment whose image features are relatively sparse compared to the dataset in Section 3.1.2. An initial reference image was obtained under a 10 N preload. Subsequent loading was applied under displacement control in incremental steps at a rate of 30 μm/s. For Sample 1, the relative nominal compressive strain increments from load steps 2 to 5 are 13.68 %, 14.89 %, 13.21 %, and 5.58 %, respectively; the applied load force increments are 71 N, 91 N, 95 N, and 83 N, respectively. For Sample 2, the corresponding strain increments from load steps 2 to 7 are 1.65 %, 2.25 %, 2.26 %, 2.79 %, 2.81 %, and 2.30 %, respectively; the applied load force increments are 23 N, 73 N, 65 N, 33 N, 30 N, and 40 N, respectively. For Sample 3, the strain increments from load steps 2 to 7 are 2.20 %, 2.26 %, 2.22 %, 2.24 %, 2.24 %, and 2.23 %; the applied load force increments are 71 N, 91 N, 95 N, and 83 N, respectively; the applied load force increments are 61 N, 51 N, 38 N, 32 N, 43 N, and 52 N, resp ectively.

**Fig. 8:**
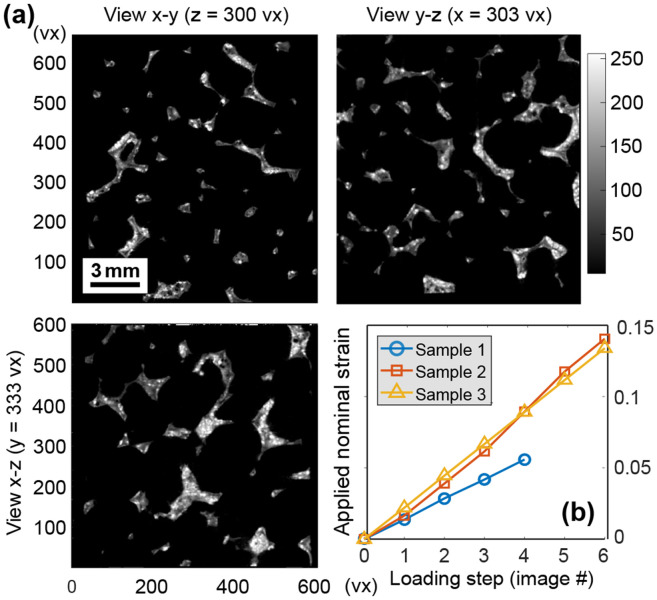
Overview of the Sriram Foam dataset images. (a) Orthogonal views (x-y, y-z, and x-z planes) of the reference volumetric image for Foam S1, revealing its porous microstructure. The horizontal and vertical axes denote spatial coordinates in voxel (vx) units. The colorbar represents grayscale intensity values of the volumetric image. (b) Applied compressive strain relative to the initial 10 N preload as a function of image acquisition step for the three tested samples. [Dataset corresponds to Section 3.1.3]

After each loading step, the sample was allowed to relax for 15 min before XCT imaging was performed to capture the deformed state. The deformation history, showing the applied incremental compressive strain at each step for the three samples, is detailed in Fig. 8(b). The applied nominal strain values are similar for both Foam S2 and Foam S3, while Foam S1 showed slightly lower values due to the differences in applied displacements. However, the displacement rates remained the same, which is more critical in polymeric samples [78].

This sparse nature is visually confirmed in the orthogonal views presented in Fig. 8(a). The images show large, interconnected voids separated by relatively thin solid struts. The high porosity means that large portions of the volume contain no grayscale information, forcing DVC algorithms to rely solely on tracking the features on the solid phase, which can be difficult if subsets contain too much empty space.

This structural sparseness is quantified by the autocorrelation analysis (see the Supplementary Mate rial Fig. S4). The slow decay of the mean autocorrelation curve is a direct result of the large average size of the foam’s structural elements (the voids and struts). This yields a characteristic mesoscale length (10 % $\bar{AC}$ length) of approximately 31 vx (averaged across the three samples), which is substantially larger than that observed in the denser foam.

In dense materials, feature size is governed by fine-scale texture, whereas in highly porous or lattice-like structures, it is dominated by void size and structural spacing. Therefore, variations in feature size should be interpreted as reflecting differences in structural length scales and imaging conditions. The observed variation in feature size across samples (1/e $\bar{AC}$ length 13.4–15.1 vx) is primarily attributed to differences in pore size distribution and strut thickness among the foam specimens, rather than data quality. In highly porous open-cell structures, the autocorrelation length is dominated by the characteristic spacing between voids and solid struts. Consequently, larger pores and more widely spaced structural elements lead to larger correlation lengths. A large correlation length also implies that a larger DVC subset (interrogation window) is necessary to capture a sufficient amount of unique texture for a stable and accurate measurement. Furthermore, the narrow standard deviation band indicates that the microstructure, while sparse, exhibits minimal directional dependence. The complete dataset parameters are summarized in Table 4.

#### 3.1.4 Wood: orthotropic and highly aligned fiber composites (Chen et al. [79])

**Fig. 9:**
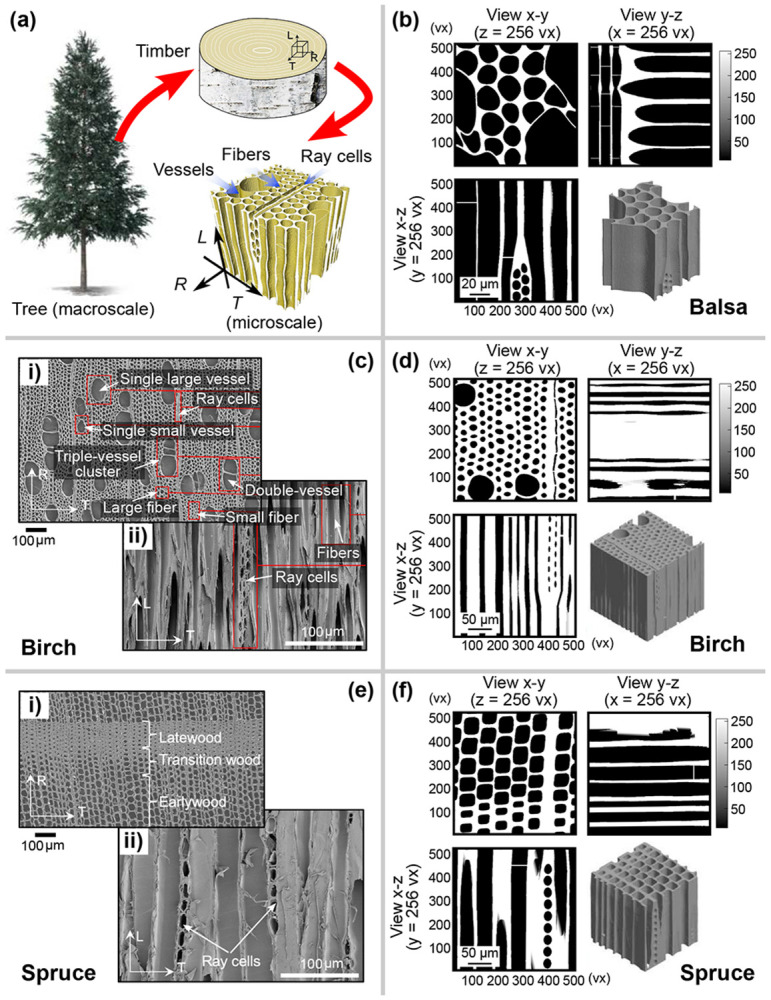
Overview of the synthetically generated wood datasets. (a) Hierarchical hardwood structure from macroscale to microscale. The typical hardwood microstructure comprises fibers and large vessels oriented in longitudinal (“L”) direction, and ray cells in radial (“R”) orientation and vertical to tangential (“T”) orientation. (b,d,f) Orthogonal views of the synthetic reference volumetric image of different wood structures. The horizontal and vertical axes denote spatial coordinates in voxel (vx) units. The colorbar represents grayscale intensity values of the volumetric image. (c,f) Scanning electron micrographs in R-T and L-T planes for Birch and Spruce (adapted from [79]), where (i) and (ii) display different wood material microstructures. [Dataset corresponds to Section 3.1.4]

This dataset includes numerically generated realistic 3D microstructure models of three typical cellular biocomposites, specifically, Birch (hardwood, high density with relatively small fibers), balsa (hardwood, low-density with relatively large fibers), and Spruce (softwood) structures. The typical hardwood microstructure comprises fibers and large vessels oriented in the longitudinal (“L”) direction, and ray cells in the radial (“R”) orientation and vertical to tangential (“T”) orientation (see Fig. 9(a)). The Spruce presents a clear annual ring with obvious density variation from earlywood to latewood along the R direction. Deformation measurement of wood microstructure using DVC is challenging, since wood grains have a preferred direction along the L direction. Since fewer unique features exist in the L direction, pattern-induced bias errors are typically larger in the L direction compared with the R and T directions. These orthotropic microstructures from wood cross sections can be used to probe the efficacy of DVC codes on orthotropic materials and highly aligned composites, which remains a challenge for DVC algorithms.

A numerical framework is used to generate representative image volumes. The framework provides flexible parametric control over essential microstructural characteristics, including fiber diameter, cell wall thickness, the presence of ray cells or vessels, and overall specimen dimensions, allowing for systematic generation of diverse wood and wood-based composite architectures (e.g., native wood, wood–polymer composites). These synthetic images are almost binary. Although only a few datasets are included here, the method is computationally efficient and scalable, enabling rapid production of large datasets by controlled variation of a small set of physically meaningful microstructural parameters.

Figure 9(b,d,f) shows the cross-section images of the numerically generated 3D wood microstructure models of three different types of wood microstructures in longitudinal (“L”), radial (“R”), and tangential (“T”) orientations. The respective 3D renderings are shown in the bottom right of each subfigure. Fig. 9(c,e) presents the corresponding scanning electron micrographs for Birch and Spruce woods with typical microstructure labeled. The dataset only includes the reference volumetric images without any applied deformation. The characteristic features are summarized in Table 5.

The autocorrelation functions of the three wood species (see Supplementary Material Fig. S5) reveal distinct structural characteristics. Balsa exhibits the largest characteristic mesoscale size (10 % $\bar{AC}$ length of 42.5 vx), Birch the smallest (15.1 vx) (Birch here refers to the S1 variant in the dataset repository), and Spruce is intermediate (22.1 vx). All three species display wide standard deviation bands consistent with the expected strong directional dependence of wood microstructures, and the Birch curve exhibits oscillations indicative of quasi-periodic cellular organization.

This data provides a unique combination of an anisotropic inherent pattern with a simulation-based approach. This approach allows the generated volumes to be inherently noise-free, thereby enabling a controlled and unbiased evaluation of DVC algorithms for specific microstructures without confounding imaging artifacts. Readers can generate additional wood microstructures using the open-source code on GitHub (https://github.com/BinChenOPEN/Wood-Microstructure-Modeling).

#### 3.1.5 Granular materials from the “Software for Practical Analysis of Materials (SPAM)” (O. Stamati, E. Andò et al. [80])

This dataset originates from the “Software for Practical Analysis of Materials (SPAM)” [80] and comprises five granular material sub-datasets, each obtained using either XCT or NeXT-Grenoble [81], a simultaneous neutron and X-ray scanner at the Institut Laue-Langevin in Grenoble. NeXT-Grenoble is designed to capture the deformation behavior of granular materials under controlled conditions. These datasets encompass various motion modes, including rigid body motion and triaxial compression.

The SPAM Sandstone dataset consists of XCT scans of a notched sandstone sample, recorded before and after a measurable plastic strain increment (as well as a rigid body movement between scans) [82]. Similarly, the SPAM Soil dataset, also acquired through XCT, records the triaxial compression of a small sample of Martian Simulant soil, providing valuable data for numerical modeling of soil behavior [83]. The SPAM Concrete S1 and S2 datasets present XCT scans of a micro-concrete cylindrical sample, offering insights into its structural response under stress [84].

In addition to these XCT datasets, the SPAM Sand S2 (SandNX) dataset was obtained using NeXT-Grenoble, which provides both neutron and XCT imaging [85]. Neutron computed tomography (NCT) imaging is particularly sensitive to hydrogen-rich fluids within the sample, whereas XCT is more suited for visualizing the solid framework. This dataset has been downscaled to match the resolution of the XCT images for comparative analysis. Finally, the SPAM Concrete S3 (YehyaConcreteNX) dataset, also acquired via NeXT-Grenoble [86], features low-permeability concrete previously used in studies of fluid flow and pressure gradient analysis using neutron imaging.

**Fig. 10:**
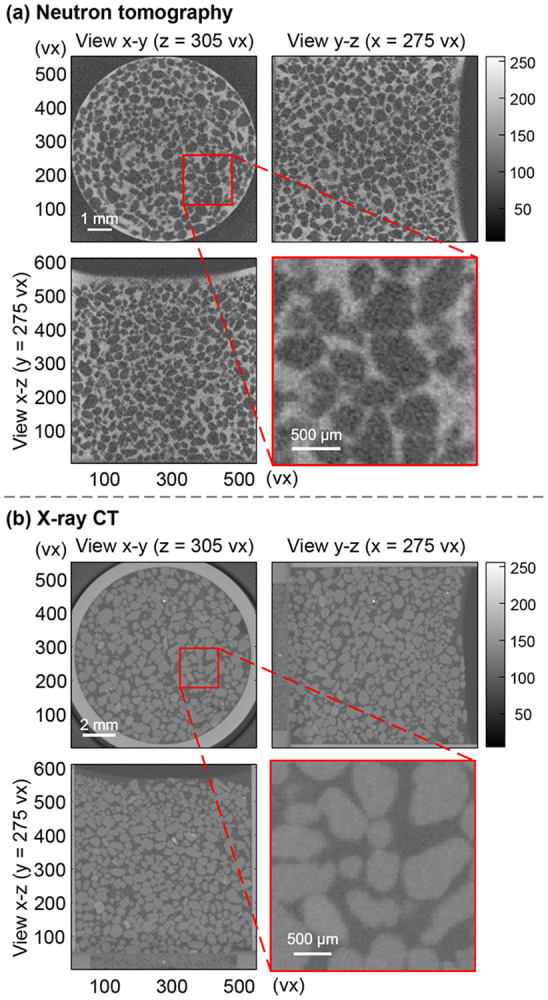
Orthogonal views of the reference volumetric image for SPAM “Sand” datasets: (a) NCT image (Sand S2); (b) XCT image (Sand S1). The horizontal and vertical axes denote spatial coordinates in voxel (vx) units. The colorbar represents grayscale intensity values of the volumetric image. [Dataset corresponds to Section 3.1.5]

**Fig. 11:**
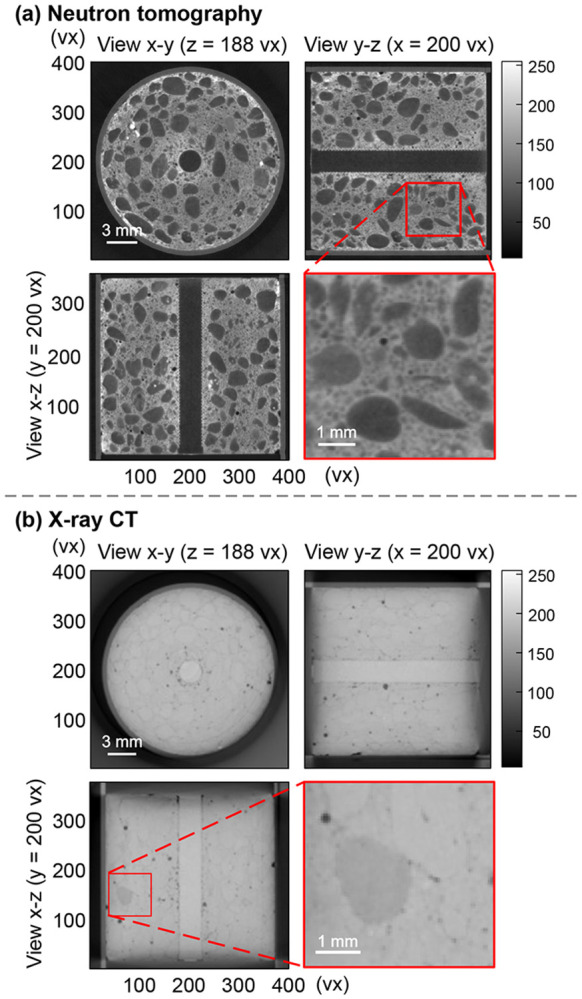
Orthogonal views of the reference volumetric image for SPAM “Concrete” datasets: (a) NCT image (Concrete S3); (b) XCT image (Concrete S2). The horizontal and vertical axes denote spatial coordinates in voxel (vx) units. The colorbar represents grayscale intensity values of the volumetric image. [Dataset corresponds to Section 3.1.5]

Representative orthogonal slice views of the SPAM Sand S1 and Sand S2 datasets are shown in Fig. 10, where both XCT and NCT images of the same specimen are displayed. Representative orthogonal slice views of the SPAM Concrete S1 and S3 datasets are shown in Fig. 11. The autocorrelation analysis is provided in the Supplementary Material Fig. S6.

A summary of the key imaging and analysis parameters for the five SPAM datasets is provided in Table 6. A notable observation is the substantial separation between the feature-scale (1/e autocorrelation length) and mesoscale (10 % autocorrelation length) for Dataset Concrete S2, where the mesoscale length is approximately 35.6 vx, while the feature size remains near 6.2 vx. This disparity arises from the presence of multi-scale structural heterogeneity in the concrete specimen. Specifically, the feature-scale texture governed by local variations within the solid matrix, whereas the long-tail decay of the autocorrelation function reflects large-scale spatial organization, such as pore clusters, cracks, or low-frequency density variations.

For DVC analysis, this implies that a single characteristic length scale is insufficient to fully describe image texture. The large mesoscale length suggests that larger subset sizes may be required to capture global structural correlations, while the smaller feature size indicates that local displacement tracking remains feasible at finer scales. Thus, the discrepancy between feature and mesoscale sizes reflects the inherently multi-scale nature of the material and provides complementary insight into both the material and the application of DVC. Additionally, the dataset includes both rigid-body displacements and mechanically induced deformation processes, making them valuable benchmark dataset for evaluating DVC performance as well as for studying microstructural evolution in geomaterials and concrete. The inclusion of both XCT and NCT, with similar materials, offers a unique comparison of using DVC with these techniques.

#### 3.1.6 Vat photo-polymer built metamaterial compression (O. Kafka et al.)

Vat photopolymerization was used to fabricate compression test specimens consisting of two integrated platens connected by a cylindrically mapped triply periodic minimal surface (TPMS) lattice structure with a nominal wall thickness of 400 μm. These specimens were printed using an LCD-based additive manufacturing system with a pixel size of 40 μm. The printing material was PR-48 (Arkema, Boulder, CO) modified with 0.4 mass% Phenylbis(2,4,6-trimethylbenzoyl) phosphineoxide photoinitiator. The layer exposure time was 4.25 s with a nominal optical power of 2 mW/cm^2^.

**Fig. 12:**
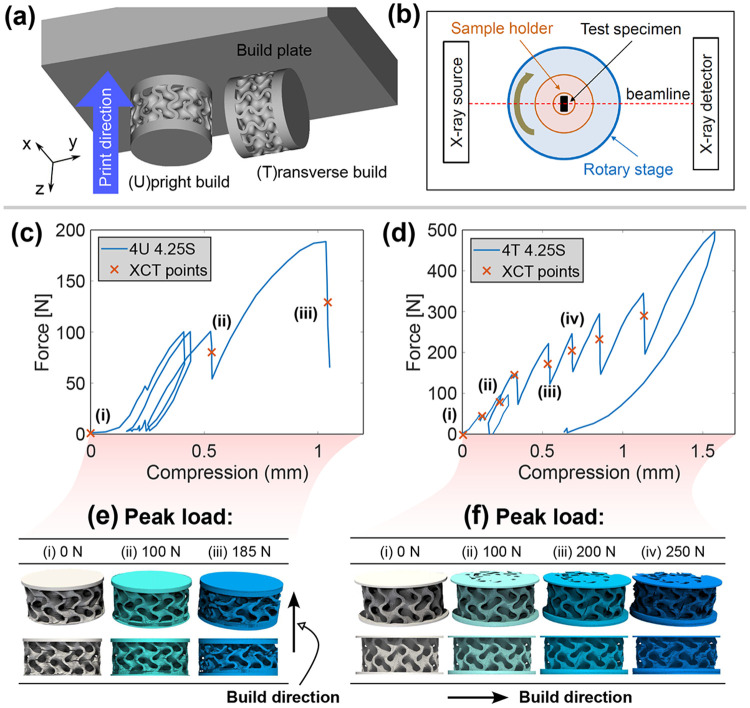
Experimental setup and interrupted in-situ compression testing of the additively manufactured metamaterial specimens. (a) Schematic of the upright (U) and transverse (T) build orientations relative to the print direction. (b) Diagram of the lab-scale X-ray computed tomography (XCT) setup used for imaging. (c,d) Force-compression curves for the upright and transverse specimens, respectively, with orange markers indicating the load points where XCT scans were acquired. (e,f) 3D volumetric renderings of the specimens at each corresponding load step, visualizing the progression of deformation under compression. [Dataset corresponds to Section 3.1.6]

The specimens were printed in *Upright* (build direction along the primary axis, denoted as “U”) and *Transverse* (build direction perpendicular to the primary axis, denoted as “T”) configurations, shown in Fig. 12(a). Mechanical testing was conducted using an interrupted *in-situ* compression approach combined with laboratory-scale XCT, using the same instrument and load stage as used in Section 3.1.2.

For the transverse configuration, loading was stopped at 50 N increments for successive 3D image collection, from 0 N to 350 N. For the upright configuration, images were taken at 0 N, 100 N, and 185 N load levels (see Fig. 12(f) and (e), respectively).

**Fig. 13:**
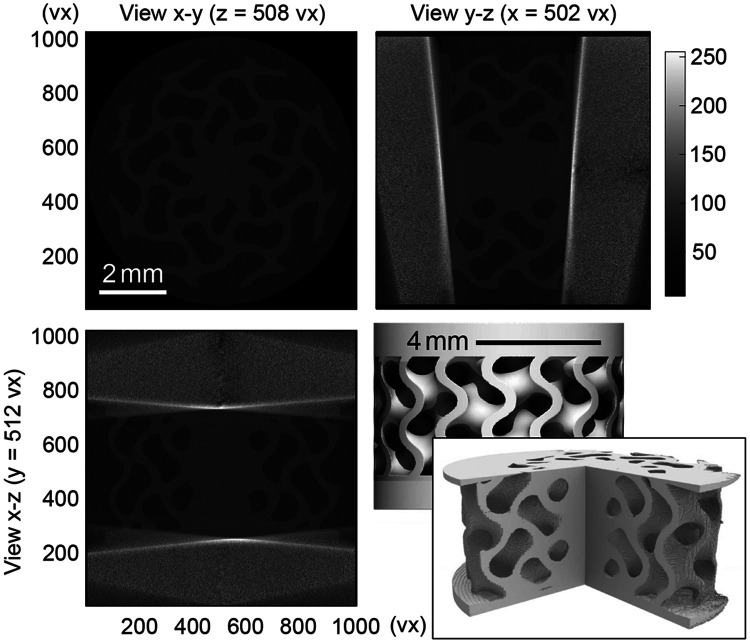
Orthogonal XCT views and 3D renderings of the additively manufactured polymer metamaterial specimen. The images display the complex triply-periodic minimal surface (TPMS) lattice structure situated between two integrated platens for compression testing. The horizontal and vertical axes denote spatial coordinates in voxel (vx) units. The colorbar represents grayscale intensity values of the volumetric image. [Dataset corresponds to Section 3.1.6]

XCT images were collected using 50 kV and 79 μA power settings, at 9.00 μm voxel edge length with exposure time 2.8 s and 1401 projections for the T condition. Settings for the U condition were slightly different, with 8.25 μm voxel edge length, 3.2 s exposure time, and 2401 projections. For both, camera binning of 2 × 2 resulted in 1004 × 1024 pixel projection images and a final image shape of 1004 vx wide by 1024 vx tall by 1016 vx deep. Reconstruction of the 3D images was achieved using the built-in software (Zeiss Reconstructor version 14) with a beam hardening constant of 0 for t and 0.12 for u. Sixteen-bit grayscale TIFF image stacks were exported and processed through local means denoising and hysteresis thresholding to achieve binary (black-and-white) images, using the IMPPY3D [56] software package. Orthogonal views of the upright-built sample are shown in Fig. 13, and the corresponding autocorrelation analysis is provided in the Supplementary Material Fig. S7.

A summary of the key parameters for the two Vat-photopolymer metamaterial datasets is provided in Table 7.

The metamaterial nature of the specimens used for these images provides a non-random repeating unit cell, which could challenge some DVC codes differently than other anisotropic datasets. Because of the relatively well-controlled nature of the struts and multiple orientations of loading it can help provide insight into the effect of loading boundary conditions and assumptions made about behaviors within the inherent pattern.

#### 3.1.7 Polymeric specimens with an open-cell Kelvin lattice structure (A. Shaikeea et al.)

Polymeric specimens with an open-cell Kelvin lattice architecture were fabricated using stereolithography (SLA). Each specimen consisted of a periodic lattice with nine unit cells along each direction and a nominal strut diameter of 0.6 mm. The parent material was a photosensitive grey resin (Grey V4, Formlabs), and printing was performed using a Formlabs Form 3 printer with a layer thickness of 50 μm.

Following fabrication, the specimens were washed in isopropyl alcohol for 10 min to remove uncured resin and subsequently UV-cured for 60 min to complete the polymerization process. After curing, all samples were stored in a dark enclosure to prevent additional unintended exposure and over-curing. Mechanical testing was conducted within one month of fabrication to ensure consistent material properties.

A custom loading rig was designed to measure both axial load and displacement during compression testing inside the XCT scanner, allowing 3D tomographic imaging under load. The specimens were compressed at a nominal strain rate of 10^−4^ s^−1^, with loading paused at displacements of 2 mm, 4 mm, and 5 mm to acquire scans. The initial undeformed state was also scanned for reference. The 3D images were obtained using a Nikon XTH 225 ST system with a mean voxel size of 40 μm × 40 μm × 40 μm. The electrode excitation voltage in the X-ray source was set to 65 kV and the current to 359 μA. No external filter was applied during image acquisition. About 3000 projections were captured with an exposure time of 1 s per projection. The acquired images were reconstructed using Nikon software with minimal beam-hardening correction.

**Fig. 14:**
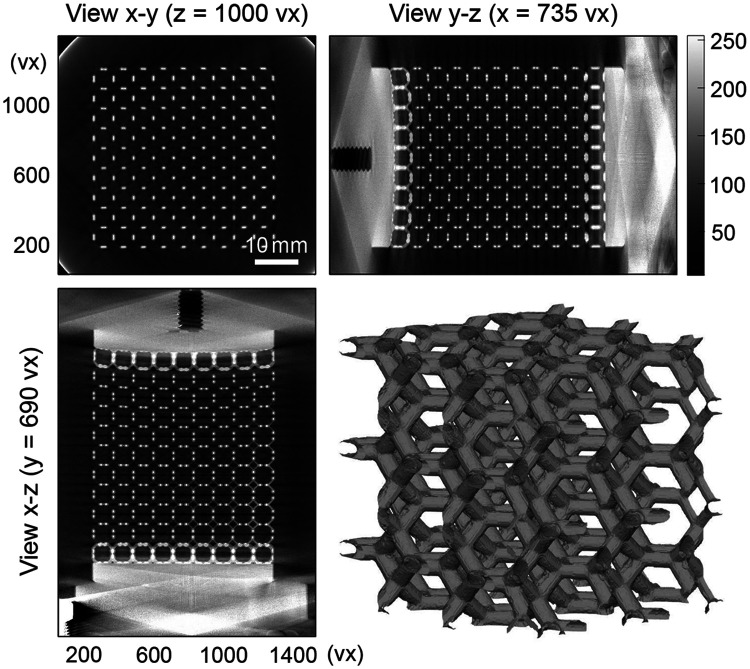
Orthogonal views and 3D rendering of the reference volumetric image for the polymeric specimens with an open-cell Kelvin lattice structure. The horizontal and vertical axes denote spatial coordinates in voxel (vx) units. The colorbar represents grayscale intensity values of the volumetric image. [Dataset corresponds to Section 3.1.7]

Representative orthogonal views of the reconstructed reference volume are shown in Fig. 14, illustrating the repeating Kelvin lattice architecture. The corresponding autocorrelation analysis is provided in the Supplementary Material Fig. S8. A summary of the key material, imaging, and analysis parameters for this dataset is provided in Table 8. Similarly to the distinction between the dense polymer foam and the sparse foam, this data is comparable to the dense 3D-printed XCT dataset above, but has unit cells on a much larger length scale and much less dense features while being isotropic. This regular, isotropic, low-density pattern is an important use-case to test.

### 3.2 Optical Microscopy

#### 3.2.1 Synthetic fluorescent microscopy with embedded fluorescent microparticles([14, 15, 87, 88])

This dataset aims to simulate a common mode of experimental soft-material imaging, where fluorescent beads are embedded within transparent matrices to create trackable volumetric features for displacement analysis. For example, in polyacrylamide (PAAm) hydrogels, different-colored fluorescent beads have been incorporated into the precursor solution prior to gelation [14, 15, 50, 88]. Patel et al. [87] reported the use of carboxylate-modified microspheres (FluoSpheres) at final concentrations of 14 % and 2 % for 1.0 μm red fluorescent beads (580 nm excitation/605 nm emission) and 2.0 μm blue-green fluorescent beads (430 nm/465 nm), respectively.

To generate synthetic volumetric datasets that mimic beads embedded in soft materials, N-dimensional Poisson-disc sampling was used to randomly distribute points in space while enforcing a minimum separation distance between neighboring particles. At these locations, image intensity is modified by interpolating a dot convolved with the point spread function of the optics into the image. This approach produces spatially realistic bead patterns suitable for synthetic generation of microscopy images and controlled DVC benchmarking.

**Fig. 15:**
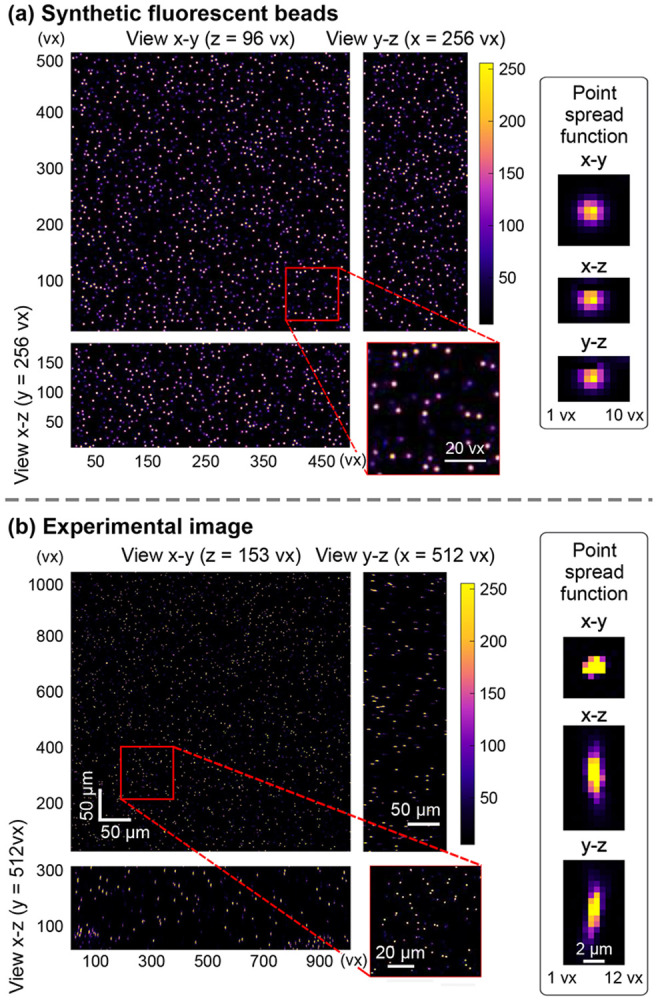
Orthogonal views of the reference volumetric image for the polyacrylamide hydrogel solution incorporating synthetic fluorescence beads. (a) Synthetic volume generated for the DVC Challenge 2.0 dataset. (b) Experimental confocal image from Patel et al. [87], shown for visual comparison only (not part of this dataset). The horizontal and vertical axes denote spatial coordinates in voxel (vx) units. The colorbar represents grayscale intensity values of the volumetric image.

Representative orthogonal views of the synthetic reference volume are shown in Fig. 15(a). For visual comparison, an experimental confocal fluorescence microscopy image of fluorescent microspheres embedded in a polyacrylamide hydrogel, originally acquired by Patel et al. [87], is shown in Fig. 15(b). Note that this experimental image is not part of the DVC Challenge 2.0 dataset; it is included solely to illustrate the visual fidelity of the synthetically generated volume relative to real microscopy data.

A summary of the key parameters for this synthetic bead dataset is provided in Table 9. The autocorrelation analysis of the reference volume is shown in Supplementary Material Fig. S9. The sinusoidal displacement field is defined as a spatially varying sinusoidal function,

(12) u(x)=A(x)sin(2πx∕λ(x)),

where the amplitude A(x) and wavelength λ(x) decrease linearly across the image domain, similarly to the DIC Challenge Sample 14 test case [43]. In the synthetic dataset, the spatial wavelength varies from approximately 200 vx to 10 vx, producing a displacement field that contains both large-scale and high-spatial-frequency deformation components.

For the rigid translation case, a constant displacement vector was applied uniformly to all particle coordinates in one of the three principal directions {x, y, z}. The applied translation magnitude ranged from (0 to 1) voxel with increments of 0.1 vx, corresponding to a maximum rigid shift of one voxel of the image volume [50]. This produces a rigid-body translation of the entire volume without internal deformation(i.e., rigid-body zero-value). The stretch deformation is described by a homogeneous normal stretch along one or more principal axes, characterized by a deformation gradient tensor:

(13) $$F=\left[\begin{array}{ccc} \lambda_{1} & 0 & 0\\ 0 & \lambda_{2} & 0\\ 0 & 0 & \lambda_{3} \end{array}\right],$$

where $\lambda_{i}\;(i=1,\;2,\;3)$ are the principal stretches. In the tests, uniaxial stretch was applied along the x-direction, with stretch ratios varying from λ = 1.0 to 1.3 in increments of 0.05, while the y- and z-directions remained unchanged. Particle coordinates were scaled along the stretch direction, resulting in a uniform strain field throughout the volume. In the rotation case, particles were rotated about a specified axis passing through the center of the volume using a rigid-body rotation matrix. The applied rotation angles ranged from 0° to 25° in increments of 5°. The displacement magnitude increases linearly with the distance from the rotation axis.

This synthetic dataset is used to evaluate methods for measuring three-dimensional deformations in nominally transparent materials using confocal microscopy. A synthetic dataset provides a known ground truth for the deformation, which can be quite helpful in evaluating code performance under various deformation conditions and nominal noise environments. The MATLAB implementation of the Poisson-disc sampling routine is publicly available on GitHub at https://github.com/mohakpatel/Poisson-Disc-Sampling.

#### 3.2.2 Experimental PAAm hydrogel under uniaxial tension (L. Summey et al. [89])

This dataset consists of a series of volumetric images of a PAAm hydrogel containing a small volume fraction of 1 μm-diameter fluorescent polystyrene particles added prior to crosslinking [89]. The embedded particles serve as trackable volumetric features for DVC analysis.

**Fig. 16:**
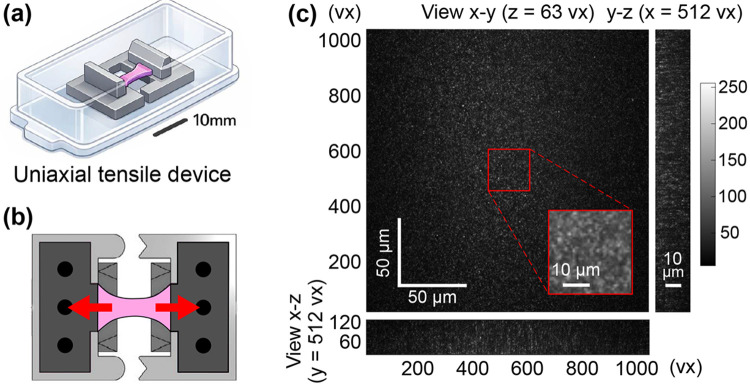
Setup diagram and orthogonal views for the Franck Polyacrylamide dataset. (a-b) Schematic of the uniaxial tensile loading configuration (adapted from [89]). (c) Orthogonal views of the reference volumetric image captured by confocal microscopy. The horizontal and vertical axes denote spatial coordinates in voxel (vx) units. The colorbar represents grayscale intensity values of the volumetric image. [Dataset corresponds to Section 3.2.2]

The specimen was subjected to stepwise uniaxial tensile loading, and three-dimensional images were captured using multiphoton microscopy with a 20× objective. To minimize transient motion and viscoelastic relaxation effects during imaging, each volumetric image stack was collected approximately 30 seconds after completion of each loading increment. Representative views of the specimen geometry and the imaging volume are shown in Fig. 16. Orthogonal slice views of the reference image are shown in Fig. 16(c).

The autocorrelation analysis of the reference volume is shown in Supplementary Material Fig. S10, where the spatial correlation decays with increasing separation distance. The imaging and analysis parameters for this dataset are summarized in Table 10. The dataset contains 28 volumetric stacks acquired during uniaxial loading. Images were collected with a voxel size of 0.200 μm × 0.200 μm × 1.075 μm, reflecting the anisotropic sampling typical of optical sectioning microscopy. The feature-scale and mesoscale autocorrelation lengths are 1.7 vx and 8.0 vx, respectively, and the estimated displacement noise floor is approximately 0.007 vx in all three spatial directions. Overall, this dataset provides high-resolution, relatively low-noise images with multiphoton images of a hydrogel-bead system. Under the tensile deformation, the spacing between beads increases and can move out of frame, which can challenge some DVC implementations.

#### 3.2.3 Experimental sphere indentation in a hydrogel (J. Yang et al. [50])

This dataset was obtained from a sphere indentation experiment performed on a PAAm hydrogel [50]. PAAm hydrogels are often used as model soft materials because of their hyperelastic mechanical response and optical transparency. In this experiment, 1 μm fluorescent beads were embedded within the hydrogel and served as the information carriers, or speckle features, for DVC tracking.

**Fig. 17:**
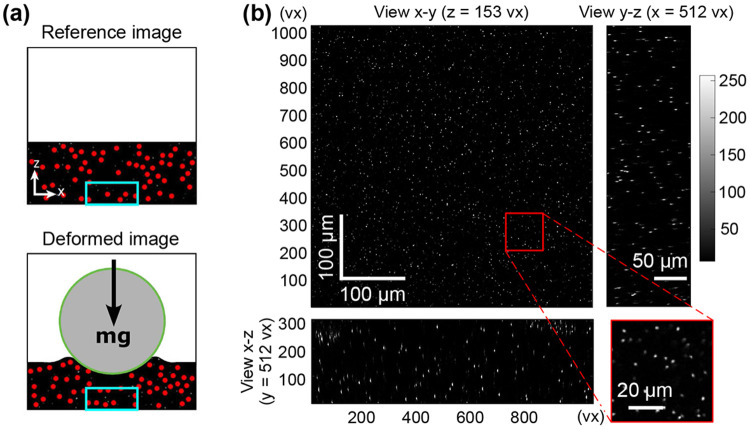
Overview of the hydrogel indentation experiment. (a) Experimental schematic of the indentation-induced deformation in a polyacrylamide hydrogel, illustrating the reference and loaded states. (b) Orthogonal views of the reference 3D image volume captured by confocal microscopy. The embedded fluorescent beads provide high-contrast features for displacement tracking, as highlighted in the magnified inset. The horizontal and vertical axes denote spatial coordinates in voxel (vx) units. The colorbar represents grayscale intensity values of the volumetric image. [Dataset corresponds to Section 3.2.3]

High-resolution volumetric images were acquired using confocal microscopy with a 25×/1.15NA water immersion objective before and after indentation, resulting in two image stacks: an undeformed reference image and a deformed image after loading. Representative orthogonal views of the stress-free reference volume are shown in Fig. 17. Each bright spot corresponds to an individual embedded fluorescent bead. The average image diameter of a single bead is approximately 5 vx.

Both the reference and deformed image stacks have dimensions of 1024 vx × 1024 vx × 306 vx, corresponding to a physical domain size of approximately 430 μm × 430 μm × 130 μm. The dataset parameters, including imaging resolution, characteristic autocorrelation lengths, and representative DVC noise-floor settings, are summarized in Table 11. The autocorrelation analysis of the reference volume is shown in Supplementary Material Fig. S11.

This dataset represents an example of strongly inhomogeneous deformation in a soft material subjected to localized mechanical loading imaged with conventional confocal microscopy. This inhomogeouns, but well-controlled and analytically modelable (e.g., [50] provides an effective test case for many types of DVC codes.

#### 3.2.4 Synthetic Collagen Fiber Network (M. Sarkar and J. Notbohm [90])

This synthetic dataset describes a three-dimensional model of a fiber network, simulating a 2 mg/mL collagen I matrix, undergoing general shear deformation. The model preserved the structural fidelity of collagen I matrices by using an optimization algorithm to randomly assemble fibers in three dimensions [91-93], achieving a desired average fiber length with an average of approximately three fibers connecting at each node. Fibers were modeled as beams welded at nodes to transfer forces and moments.

A shear deformation was applied to the network model using finite element software to solve for the deformed states where fibers experienced stretching, bending, and buckling. As a result, the displacements at each node in the fiber network, obtained from the finite element simulation, serve as ground-truth data.

**Fig. 18:**
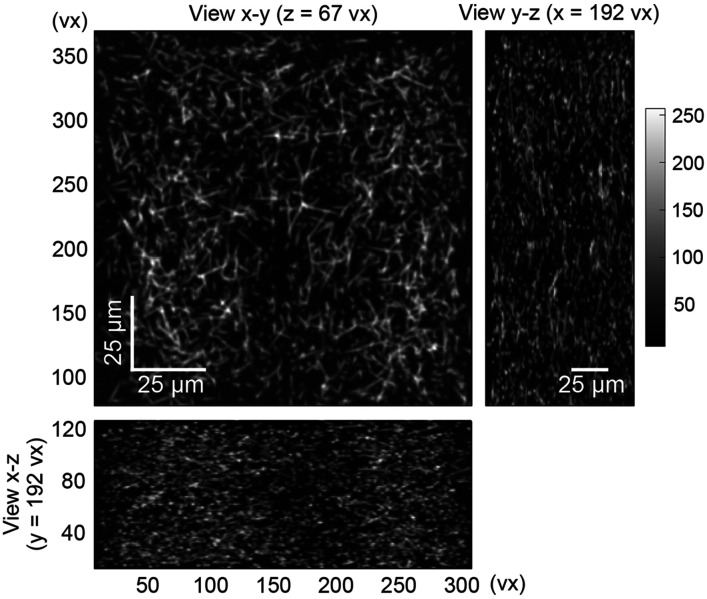
Orthogonal views of the reference volumetric image for the synthetic collagen fiber network. The horizontal and vertical axes denote spatial coordinates in voxel (vx) units. The colorbar represents grayscale intensity values of the volumetric image. [Dataset corresponds to Section 3.2.4]

Synthetic image stacks representing both the undeformed and deformed states were generated by convolving the three-dimensional fiber network with the point spread function of a confocal microscope [90]. These synthetic images mimic z-stack imaging of collagen networks acquired with a confocal microscope equipped with a 20× air objective with a numerical aperture of 0.7 and an illumination wavelength of 525 nm. In the resulting images, the fibers themselves act as the high-contrast structural features used for DVC tracking. Representative orthogonal views of the synthetic collagen network are shown in Fig. 18. DVC results can be compared directly with the provided finite element ground truth displacement field, which is available in separate spreadsheets indicating the position and displacement at each node.

The results for this dataset are summarized in Table 12. The autocorrelation analysis of the reference volume is shown in Supplementary Material Fig. S12. DVC results can be compared directly with the provided finite element ground truth displacement field, which is available in separate spreadsheets with data for the position and displacement at each node. The fibrous material is a useful test case since it mixes elements of random patterns on longer length scales with challenging local isotropy when using the inherent texture of the fibers. The code to generate the fiber networks and synthetic images is available on GitHub at https://github.com/jknotbohm/fiber_network_model and https://github.com/jknotbohm/image_generator, respectively. These can be used to generate additional volumetric images under different boundary conditions, facilitating further comparisons between DVC measurements.

#### 3.2.5 Experimental Collagen Network with A Contracting Hydrogel Inclusion (B. Burkel and J. Notbohm [94])

This experimental dataset consists of confocal microscopy images of a three-dimensional collagen fiber network containing a centrally located spherical hydrogel inclusion, i.e., a spherical poly(*N*-isopropylacrylamide) (PNIPAAm) particle that contracts under thermal stimulation [94]. Fibers are made of collagen I at a concentration of 2 mg/mL suspended in a fluid environment. Collagen networks of this type are widely used in mechanobiology to study how cells sense and respond to the physical structure and mechanics of fibrous extracellular environments. Typical collagen specimens in this dataset are a few millimeters in diameter and approximately 0.5 mm thick. The collagen fibers were fluorescently labeled to enable imaging with confocal microscopy.

The spherical inclusion in the center is made of PNIPAAm, a thermoresponsive hydrogel that undergoes a phase transition, causing a dramatic decrease in volume when heated. Because the collagen fibers were covalently attached to the spherical inclusion, contraction of the sphere induced inward pulling of the surrounding fiber network. Control experiments without embedded hydrogel spheres verified that temperature changes alone caused negligible displacements and did not measurably alter the stiffness of the collagen network [94-96]. Unique features of this experiment are that the contracting spheres produce large, spatially varying displacements and strains on cellular length scales while avoiding many of the gripping challenges that typically arise in soft-material testing, providing a relatively uniform, spherically symmetric spatial gradient.

A reference image stack was first acquired at a nominal temperature of 22 °C. The temperature was then increased, followed by roughly 20 min for temperature equilibration, and another image stack was acquired. This process was repeated three times, providing a total of five image stacks: the first being an undeformed reference, and the remaining four being at increasing levels of sphere contraction. Imaging was performed using a spinning disk confocal microscope, as described in Ref [94]. Temperature changes introduced some rigid-body drift between image stacks, and this motion can be corrected during analysis by subtracting the rigid-body motion from the resolved displacement fields.

**Fig. 19:**
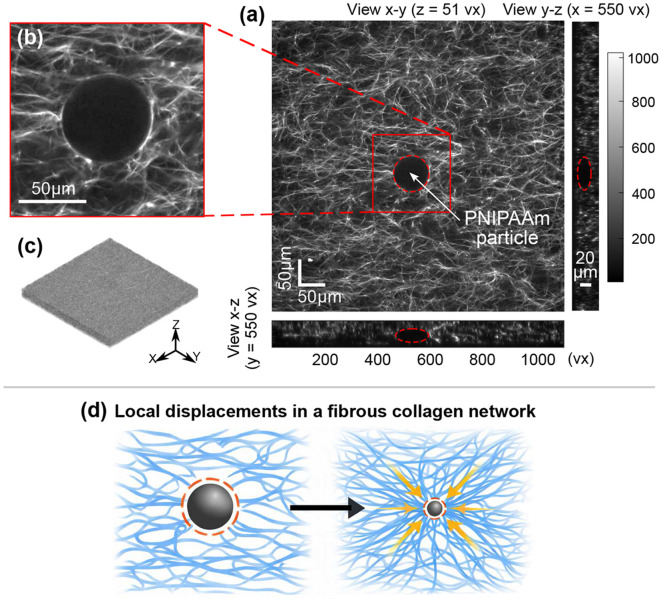
Overview of the fibrous material inclusion experiment. (a) Orthogonal views (x–y, x-z, and y–z) of the reference volumetric image of a fibrous collagen network containing a PNIPAAm particle, acquired via confocal microscopy. The red box highlights the region of interest, with a zoomed-in view shown in (b). The spherical inclusion appears non-circular in the x–z and y–z planes due to anisotropic voxel scaling, where the pm-to-voxel ratio in the z-direction differs from that in the x- and y-directions. The horizontal and vertical axes denote spatial coordinates in voxel (vx) units. The colorbar represents grayscale intensity values of the volumetric image. The image is recorded at 10-bit depth, with a maximum grayscale value of 1023. (c) Three-dimensional rendering of the volumetric dataset. (d) Schematic illustration of local displacement fields in the collagen network induced by particle contraction. [Dataset corresponds to Section 3.2.5]

Representative orthogonal views of the undeformed reference image are shown in Fig. 19. There was no evidence of time dependence in the displacement field, which is reasonable given that temperature was allowed to stabilize for tens of minutes after being increased. The imaging and analysis parameters for this dataset are summarized in Table 13. The autocorrelation analysis (see the Supplementary Material Fig. S13) shows a wider deviation band than the synthetic collagen dataset, reflecting the reduced number of z-planes collected to save acquisition time, given the spherically symmetric boundary condition.

This dataset represents a combination of characteristics observed in previous cases, featuring a localized, well-defined strain field alongside a fibrous microstructural pattern. It also introduces a third imaging modality, spinning disk confocal microscopy, which enables high-speed volumetric imaging but may exhibit increased noise levels and imaging artifacts compared to laser-scanning confocal or multiphoton techniques.

#### 3.2.6 Multiphoton Second Harmonic Generation Microscopy of the Human Lamina Cribrosa (D. Midgett, T. D. Nguyen, et al. [17])

This dataset originates from the experimental work of Midgett et al. [17], who investigated the three-dimensional deformation of the human lamina cribrosa (LC) under controlled intraocular pressure. The experiments were performed using a custom inflation chamber with tilt-correcting gears and mounted on a Zeiss laser-scanning microscope (LSM 710 NLO). The intraocular pressure was regulated by a water column, and all testing was completed within 48 h post-mortem using eight eyes from six non-glaucoma human donors. Sample preparation followed an established protocol: extraocular tissues were removed, the optic nerve head (ONH) was sectioned flush with the sclera to expose the LC, and the posterior scleral shell was affixed to a polycarbonate ring. The anterior sclera, cornea, and intraocular contents were excised, leaving the posterior cup, which was hydrated in phosphate-buffered saline (PBS) during imaging (see [17]).

Second Harmonic Generation (SHG) laser-scanning microscopy was used to visualize the native collagen architecture of the lamina cribrosa. The imaging system employed a Coherent Chameleon Ultra II laser tuned to 780 nm and a 10× 0.45 numerical aperture (NA) Apochromat objective. The specimens were pressurized incrementally from a baseline of 0.67 kPa (5 mmHg gauge) to 1.33 kPa (10 mmHg gauge) and then to 6.0 kPa (45 mmHg gauge). At each pressure level, duplicate 2 × 2 tiled z-stacks were acquired to a depth of approximately 300 μm from the posterior surface of the lamina cribrosa, using a z-step size of 3 μm. The resulting volumetric images had in-plane resolutions (i.e., voxel-size) of approximately 2 μm to 2.5 μm and an out-of-plane spacing of 3 μm. Each z-stack acquisition took approximately 3 min to 5 min to minimize time-dependent tissue creep during imaging. The SHG image stacks were post-processed using deconvolution in Huygens Essentials, followed by three-dimensional contrast-limited adaptive histogram equalization (3D CLAHE) to enhance local contrast.

**Fig. 20:**
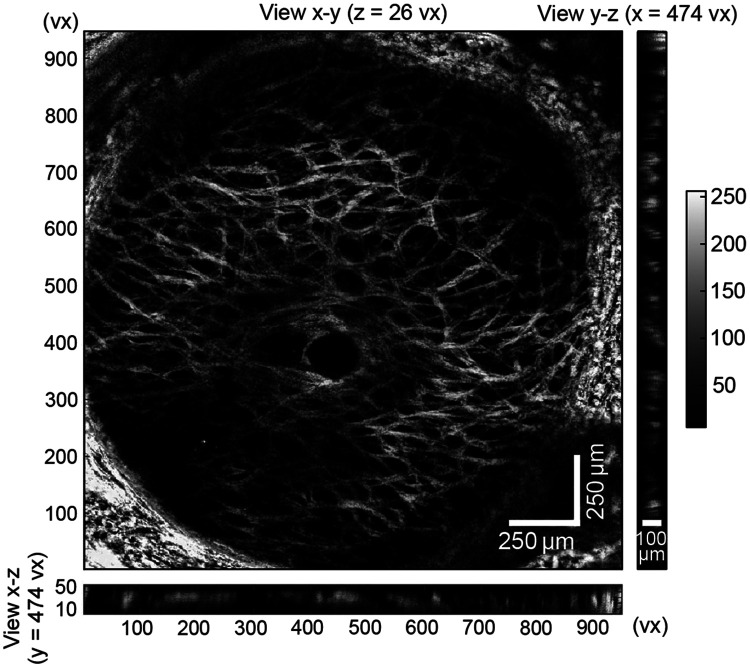
Orthogonal views of the SHG reference volume from “Run_02: HLC_0430_LE_decon_3DCL”. The volume displays the characteristic laminar collagen beam network of the human lamina cribrosa. The horizontal and vertical axes denote spatial coordinates in voxel (vx) units. The colorbar represents grayscale intensity values of the volumetric image. [Dataset corresponds to Section 3.2.6]

The complete dataset consists of six labeled subfolders, each corresponding to an independent donor test with multiple volumetric acquisitions. Within each run, both left and right eyes are included, together with their corresponding contrast-enhanced 3D CLAHE versions. As a representative example, Fig. 20 shows orthogonal views of the SHG reference volume, highlighting the characteristic laminar collagen beam network. A summary of the imaging, autocorrelation, and DVC processing parameters for this dataset is provided in Table 14. The autocorrelation analysis of the reference volume is shown in Supplementary Material Fig. S14.

This dataset introduces a fourth type of optical microscopy, SHG, for an inherently patterned biological specimen under progressively increasing deformation state. The relatively high noise floor values (Table 14) reflect the limited dynamic range of the 8-bit image depth, the lower signal-to-noise ratio typical of SHG imaging, and the coarse axial sampling (3 μm step size over only 52 *z*-slices), which particularly affects the $u_{z}$ component. These factors make this dataset a challenging but important test case for DVC algorithms applied to biological tissues.

### 3.3 Other Methods and Published DVC Datasets

#### 3.3.1 Rotating Mirror Method: Swimming Animals (M. Fu, J. O. Dabiri, et al. [97, 98])

This dataset consists of image stacks capturing both animal locomotion and the surrounding fluid flow during swimming. Each pair of images was collected using Scanning Particle Image Velocimetry [97, 98], a volumetric imaging technique in which a translating laser sheet rapidly scans through a volume of interest to sequentially illuminate slices of the volume. A high-speed camera records these image slices, encoding information about the third spatial dimension within the image time series, which can then be grouped into image stacks to create a volumetric image. The images record the light scattered from translucent animals swimming through the imaged volume and tracer particles seeded within the seawater. The time delay between images in each example is determined by the scanning frequency of the laser sheet, which, when combined with volumetric deformation, is used to calculate three-dimensional, three-component fluid velocities and animal trajectories.

**Fig. 21:**
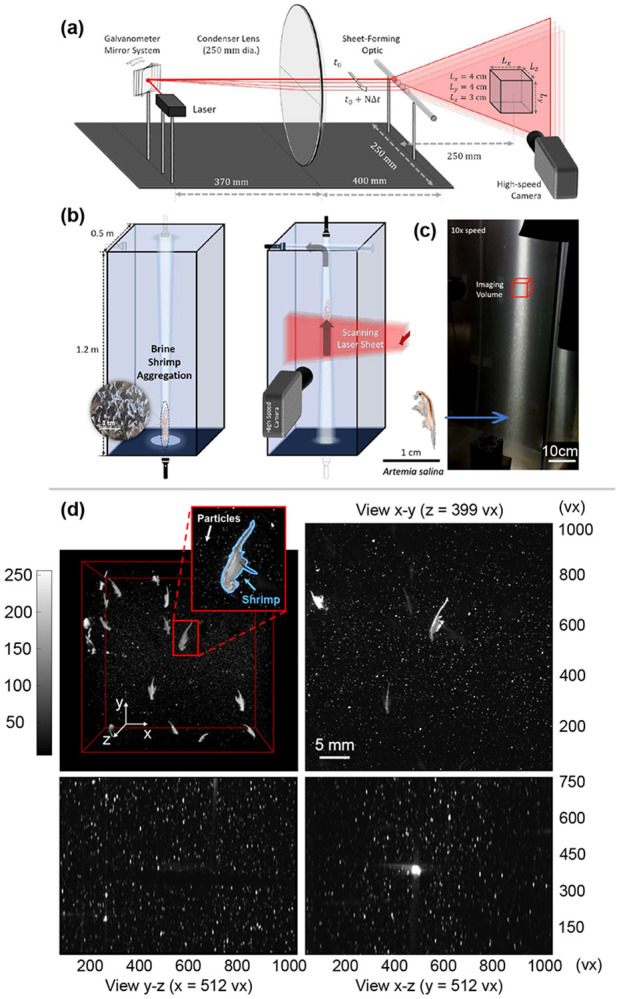
Overview of the swimming shrimp dataset. (a) Schematic of the light-sheet imaging system used to capture flow fields. (b) Experimental setup for imaging the flow field around swimming brine shrimp, illustrating the scanning laser sheet within the observation volume (adapted from [97]). (c) Example raw image showing a swimming shrimp and the surrounding flow region of interest. (d) Orthogonal views of the reference volumetric image, with an inset highlighting detected particles and the shrimp. The colorbar represents grayscale intensity values of the volumetric image. [Dataset corresponds to Section 3.3.1]

**Fig. 22:**
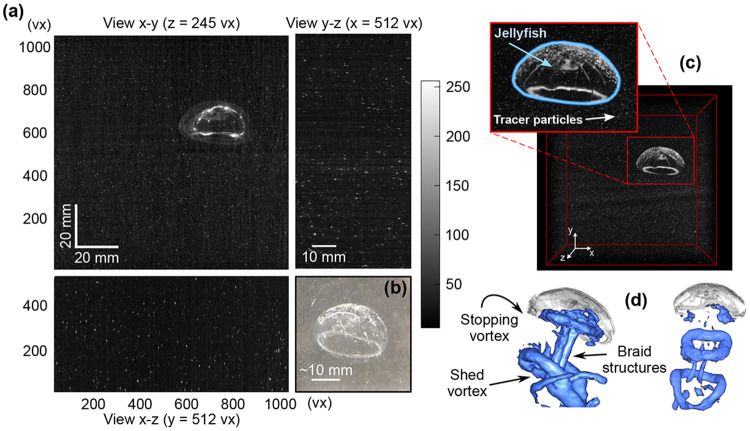
Overview of the swimming jellyfish dataset. (a) Orthogonal views of the reference volumetric image of a swimming jellyfish (*Mitrocoma cellularia*) and the surrounding flow field. The horizontal and vertical axes denote spatial coordinates in voxel (vx) units. The colorbar represents grayscale intensity values of the volumetric image. (b) Experimental photo of a jellyfish body. (c) Three-dimensional visualization of the volumetric dataset, with annotations indicating the jellyfish and tracer particles. (d) Schematic illustration of the vortex structures generated during swimming, including stopping and shed vortices. (Adapted from Ref [98]). [Dataset corresponds to Section 3.3.1]

The first dataset captures a group of approximately 40 brine shrimp (*Artemia salina*) and the surrounding flow field during an induced vertical migration event [97]. Spatial variations in scattered light intensity across the millimeter-scale animal bodies provide trackable texture for measuring shrimp displacement and deformation, while the displacement of seeded tracer particles in the surrounding fluid is used to determine the local flow velocities. The second dataset captures a single centimeter-scale jellyfish (*Mitrocoma cellularia*) swimming in a quiescent tank while shedding a vortex ring [98]. Because the jellyfish body is naturally highly transparent, a benign scattering agent, zinc oxide powder, was applied to the outer surface of the animal to enhance contrast with the surrounding fluid and improve visualization of the body deformation. The surrounding fluid was simultaneously seeded with tracer particles to enable measurement of the associated vortex-dominated flow field. Representative orthogonal views of the brine shrimp and jellyfish volumetric images are shown in Fig. 21 and Fig. 22, respectively.

The experimental parameters for all three datasets are summarized in Table 15, which reports the imaging conditions and DVC-related parameters for both the *Artemia salina* migration (Shrimp S1 and S2, corresponding to two time points analyzed with different DVC step sizes) and the *Mitrocoma* jellyfish datasets, including volumetric image dimensions, voxel resolution, temporal separation, and characteristic autocorrelation length scales. The autocorrelation analysis of the reference volume is shown in Supplementary Material Figs. S15-S16. As shown in Figs. 21-22, particles in the background appear vertically elongated in the y–z and x–z views. This effect is primarily attributed to anisotropic imaging resolution, as optical systems typically exhibit lower axial (z-direction) resolution compared to the lateral (x–y) directions. Additional contributions may arise from finite light-sheet thickness, which integrates signal over depth, as well as potential particle motion during volumetric scanning in the z-direction. Consistent with these observations, the noise floor in the x- and y-directions is approximately one order of magnitude lower than that in the z-direction (see the last three rows of Table 15).

Together, these datasets provide valuable examples of volumetric imaging in highly dynamic fluid–structure interaction problems involving deforming biological bodies and surrounding unsteady flow.

#### 3.3.2 Synthetic Magma Compression (Collaborative Computational Project in Tomographic Imaging (CCPi))

The Collaborative Computational Project in Tomographic Imaging (CCPi) (https://ccpi.ac.uk) provides an open-access repository of datasets for XCT and tomographic reconstruction research [99]. The platform hosts a diverse collection of high-quality volumetric imaging datasets obtained from a wide range of materials and applications, including engineered components, porous materials, biological samples, geological specimens, and pharmaceutical objects, some of which may be of interest to the DVC practitioner.

Although not included directly in the collection of datasets, an example DVC-related dataset from CCPi is a compression experiment on a synthetic magma analogue. The dataset consists of two volumetric image stacks: one acquired in the undeformed reference state and a second captured after compression was applied. The images were collected at the Diamond Light Source synchrotron facility using the I12 beamline, which provides high-resolution XCT. The experiment employed a bespoke thermo-mechanical loading device known as the P2R rig, designed to apply simultaneous mechanical loading and elevated temperature conditions.

**Fig. 23:**
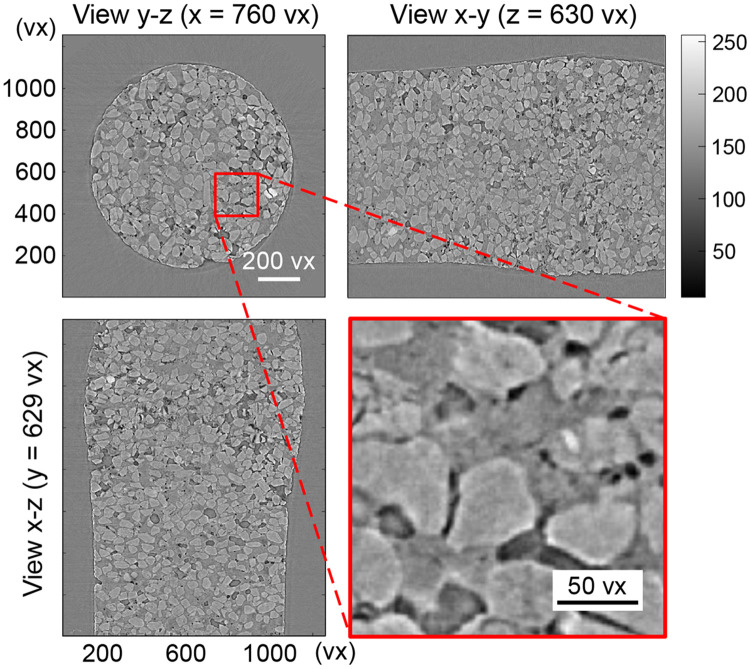
Orthogonal views of the reference volumetric image for the synthetic magma. The horizontal and vertical axes denote spatial coordinates in voxel (vx) units. The colorbar represents grayscale intensity values of the volumetric image. Length-scale bar information is not available for this dataset. [Dataset corresponds to Section 3.3.2]

Representative orthogonal views of the undeformed reference volume are shown in Fig. 23 and a summary of the imaging parameters and DVC-related metrics for this dataset is provided in Table 16. The autocorrelation analysis of the reference volume is shown in Supplementary Material Fig. S17.

#### 3.3.3 Digital Porous Media Portal (DPM [100])

The Digital Porous Media Portal (DPM) (https://digitalporousmedia.org/) is an open platform for porous media datasets, focusing on storing and sharing high-quality 2D and 3D image data and associated experimental results. The portal aims to provide a robust resource for the study of porous materials, such as rocks and soil, by offering datasets and related experimental measurements. These datasets are particularly relevant for research in environmental science, civil engineering, petroleum engineering, and geology. By promoting data sharing and collaboration, DPM aligns with the goals of DVC Challenge 2.0, offering valuable resources for the development and validation of DVC algorithms, especially in the context of porous media research.

#### 3.3.4 Optical Coherence Tomography (OCT) and Optical Coherence Tomography Angiography (OCTA) Volumetric Imaging Datasets

Optical Coherence Tomography (OCT) is a noninvasive optical imaging technique capable of acquiring high-resolution three-dimensional images of biological tissues by reconstructing depth-resolved backscattered light signals [101]. Volumetric OCT datasets are typically formed by stacking a sequence of cross-sectional scans (B-scans) into a 3D image volume. Optical Coherence Tomography Angiography (OCTA) further extends this capability by detecting temporal variations in scattered light to visualize microvascular networks without exogenous contrast agents [102]. Although OCT and OCTA datasets are primarily developed for ophthalmic imaging, disease diagnosis, and segmentation tasks, their inherent speckle patterns and structural textures can serve as natural features for DVC. Several open-access OCT/OCTA datasets are available, including OCTAVE [103], OCTA-500 [104], GAMMA [105](https://huggingface.co/datasets/ctmedtech/GAMMA), and murine OCT datasets (https://data.bris.ac.uk/data/dataset/ypfrg4sz8jwi2ehjqjubbq526), which provide volumetric images with micrometer-scale resolution suitable for studying tissue deformation, biomechanics, and optical coherence elastography. See Supplementary Material Section S4.1 for more details.

#### 3.3.5 Magnetic Resonance Imaging (MRI) Volumetric Datasets

Magnetic Resonance Imaging (MRI) is another widely used volumetric imaging modality that provides excellent soft-tissue contrast and deep tissue penetration. MRI volumes are reconstructed from multiple slice acquisitions and are typically stored in more medical-oriented formats such as NIfTI or DICOM. Although MRI datasets are primarily developed for clinical imaging, segmentation, and machine learning research, the anatomical texture and intensity variations present in MRI volumes can also provide suitable features for DVC. Several large-scale open MRI datasets are available, including the Human Connectome Project (HCP) (https://www.humanconnectome.org/), the Alzheimer’s Disease Neuroimaging Initiative (ADNI) (https://adni.loni.usc.edu/), the Brain Tumor Segmentation Challenge (BraTS) (https://www.med.upenn.edu/cbica/brats/), the IXI dataset (https://brain-development.org/ixi-dataset/), fastMRI (https://fastmri.med.nyu.edu/), and the Open Access Series of Imaging Studies (OASIS) (https://sites.wustl.edu/oasisbrains/). These datasets typically contain volumetric images with millimeter-scale voxel resolutions. Such datasets provide valuable opportunities for benchmarking DVC algorithms in biomedical contexts, including studies of tissue deformation, medical image registration, and biomechanical modeling (see Supplementary Material Section S4.2 for more details).

## 4 Noise Analysis Discussion

**Fig. 24:**
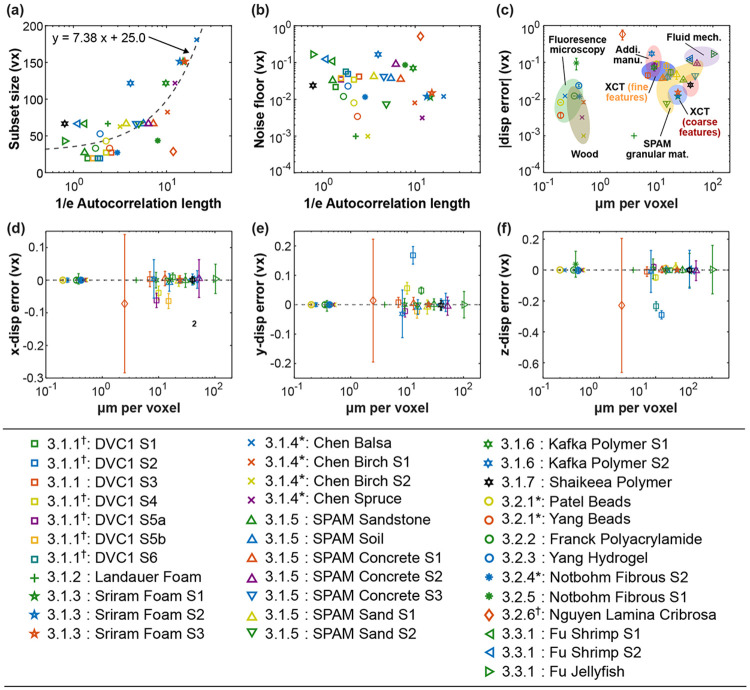
Relationship between DVC subset size, autocorrelation length, and resulting noise floor across all datasets. (a) Selected DVC subvolume window size plotted against the corresponding 1/e autocorrelation ($\bar{AC}$) length for each dataset. The approximately linear trend indicates that the subset size was systematically chosen to scale with the intrinsic feature size of each image. (b) Corresponding noise floor, as defined in Eq (2), plotted as a function of the 1/e autocorrelation length. Each data point reflects noise levels obtained using a subset size proportional to the dataset-specific $\bar{AC}$ length. (c) Displacement magnitude error ($\mu_{|u|}$) and standard deviation ($\sigma_{|u|}$) plotted on a log scale. Colored regions highlight qualitative clustering by imaging modality and material class. (d-f) Mean displacement error (bias) in the *x*-, *y*-, and *z*-directions, respectively. Symbols denote dataset-specific averages, while vertical error bars represent the corresponding random error (noise floor), quantified as the standard deviation of the measured displacements. Legend entries marked with “*” and “†” symbols denote computationally generated (synthetic) datasets and cases where the noise floor was estimated from experimentally acquired static image pairs, respectively.

Figure 24 summarizes the DVC measurement uncertainty across all datasets. In contrast to established best practices in DIC [31, 32], acquiring zero-motion (static) volumetric image pairs for DVC is often impractical. For example, collecting a single three-dimensional XCT volume may require several minutes to hours, depending on resolution and scan settings, and noise-floor characterization requires at least two consecutive scans of an undeformed specimen. Compared to near-instantaneous 2D image acquisition, this significant overhead, combined with potential drift and imaging artifacts, makes dedicated noise-floor measurements uncommon in volumetric experiments. As a result, most studies prioritize capturing the deformation process rather than acquiring repeated scans solely for noise estimation.

It is important to note that the total DVC measurement error generally consists of two primary components: (i) the systematic/random error associated with image noise [4, 51], and (ii) the image-mismatching error arising from limitations in the DVC kinematic model [52] (e.g., the chosen subset shape function not fully capturing the true underlying deformation field). The latter is often referred to as model mismatch and is known to depend on factors such as deformation gradients, subset size, and the order of the shape function. In this section, we only report the noise floor associated with image noise, and do not include additional errors arising from deformation-induced mismatching or model-form limitations.

As described in Section 2.3, the noise floor was estimated using either experimentally acquired static image pairs (denoted by “†” in the legend, available for Nguyen Lamina Cribrosa and DVC Challenge 1.0 datasets S1, S2, S4, S5a, S5b, S6) or by adding synthetic Gaussian noise to a single reference image. Synthetic datasets are denoted by “*”. Because artificial Gaussian noise does not introduce systematic bias, Fig. 24 reports only the random error (noise floor), not the bias. CCPi, Digital Porous Media Portal (DPM), OCT, and MRI datasets were not included in this analysis.

The two critical factors in the noise or scatter associated with a given DVC measurement are: (i) the local grayscale gradient, which decreases for sparse or low-contrast features and tends to increase the measurement noise floor, and (ii) the number of voxels within the subset which reduces imaging noise through statistical averaging, but is inherently tied to spatial resolution [44]. In light of this, to ensure a meaningful comparison of the datasets, the subset size for each dataset was systematically selected to scale with its intrinsic feature size, quantified by the 1∕e autocorrelation length ($\bar{AC}$). As shown in Fig. 24(a), there exists an approximately linear relationship between the chosen subset size and the 1∕e autocorrelation length across all datasets. This design choice ensures that the noise analysis is performed under comparable, feature-resolved conditions, rather than being biased by arbitrarily chosen subset sizes. Specifically, datasets with larger 1∕e autocorrelation lengths (i.e., coarser or more sparsely distributed features) require larger subsets to capture sufficient intensity variation for reliable correlation. For example, Chen Balsa ($1∕e\bar{AC}$ ≈ 20 vx) and Sriram Foam S1 (≈ 13 vx) use subset sizes on the order of 150–180 vx, whereas datasets with fine, dense features (e.g., DVC Challenge 1.0 datasets with $1∕e\bar{AC}$ ≈ [1.5–2.5] vx) use much smaller subsets (approximately 17 vx). Although the correlation exhibits moderate scatter (linear fitting *R*^2^ ≈ 0.66), this reflects practical trade-offs such as computational cost, image quality, and user-defined parameter choices, rather than a breakdown of the underlying scaling relationship. For datasets with large autocorrelation lengths, the significantly larger subset sizes provide sufficient averaging to compensate for weaker gradients, resulting in reduced noise floors. Figure 24(b) presents the resulting noise floor as a function of the 1∕e autocorrelation length. Datasets with larger feature sizes appear to exhibit lower noise levels, which may seem counterintuitive since sparse features generally provide weaker intensity gradients for correlation. However, this test does not address the reduction in spatial resolution which is the fundamental tradeoff with increasing subset size. When interpreted together with Fig. 24(a), this trend can be understood as a direct consequence of subset-size scaling.

By isolating the analysis to the noise-based contribution to measurement uncertainty, we aim to semi-quantitatively estimate the baseline measurement sensitivity of DVC under controlled noise conditions. The additional error contribution associated with deformation-induced mismatching and spatial filtering is not considered here and would typically manifest as part of a combined error curve (e.g., the U-shaped error dependence on subset size [44, 52]), which is beyond the scope of this analysis. Supplementary Material Section S5 details the computation of displacement errors for each directional component, as well as the estimation of the displacement magnitude error. Figure 24(c) plots displacement magnitude error ($\mu_{|u|}$) and standard deviation ($\sigma_{|u|}$) plotted on a log scale with colored regions indicating qualitative clustering according to imaging modality and material class. Figure 24(d-f) display mean displacement error (bias) in the *x*-, *y*-, and *z*-directions, respectively. Overall, these results highlight that meaningful cross-dataset comparison of DVC uncertainty requires normalization with respect to feature size. When the subset size is chosen proportionally to the characteristic autocorrelation length, DVC consistently achieves sub-voxel measurement accuracy across a wide range of imaging modalities and material systems.

## 5 Conclusion

In this work, we introduce DVC Challenge 2.0, which includes a collaborative and multi-modal repository of benchmark datasets for DVC. By compiling and providing baseline analyses of a diverse collection of both experimental and computationally generated volumetric images, this effort aims to address a critical need in the community for benchmarking datasets that enable rigorous validation, comparison, and improvement of DVC algorithms. The datasets span a range of imaging modalities, including XCT, neutron imaging, confocal/multiphoton optical imaging, and other volumetric acquisition techniques. The datasets further cover a broad spectrum of material systems, including metals, wood, foams and elastomers, composites, additively manufactured materials, granular materials, biomaterials and biological tissues, as well as fluid mechanics applications. Each dataset has been characterized using a consistent analysis framework that includes feature-scale characterization through autocorrelation analysis and zero-displacement noise floor. The diversity of the datasets, including multiple imaging techniques and materials, with examples of sparse features, complex microstructures, low contrast, and large deformations, provides a comprehensive set of challenges that can help drive the advancement of next-generation DVC techniques. The open-access availability (see [46]) of these datasets aims to lower the barrier to entry for researchers and students interested in applying DVC, facilitate collaboration across disciplines, and promote the development of more robust volumetric image analysis methods.

Future work will include maintaining and expanding the repository to include additional imaging modalities and materials systems, as well as organizing further benchmarking challenges that allow systematic comparison of DVC algorithms and software implementations. These efforts will also explore comparisons with related other volumetric tracking approaches, such as three-dimensional particle tracking methods [87, 106-108]. Through continued community engagement and dataset development, the DVC Challenge series seeks to strengthen the reliability, reproducibility, and broader adoption of DVC as a powerful tool for three-dimensional full-field deformation measurement in both scientific research and engineering applications.

## Supplementary Material

This is a list of supplementary files associated with this preprint. Click to download.

• DVCChallenge2DatasetsSI20260507.pdf

## Figures and Tables

**Table 1:** Summary of autocorrelation-derived descriptors and their physical interpretations.

Descriptor	Autocorrelation function thresholds	Physical interpretation
Feature size	1/e ≈ 0.368	Characteristic size of microstructural features
Feature-scale RVE	—	Minimum volume for spatially invariant statistics
Mesoscale size	0.10	Spacing of mesoscale organization or clustering
Mesoscale RVE	—	Minimum volume to capture mesoscale organization
Far-tail diagnostic	0.01	Indicator for long-range correlation or image drift
Quasi-periodicity	—	Presence of repeated or oscillatory spatial organization
Directional dependence	—	Directional uniformity and angular variation

**Table 2:** Summary of parameters for the seven XCT datasets in the DVC Challenge 1.0. [Dataset corresponds to Section 3.1.1]

Parameter	Unit	DVC1 S1	DVC1 S2	DVC1 S3	DVC1 S4	DVC1 S5a	DVC1 S5b	DVC1 S6
Material		Syntactic foam						
Imaging modality		Micro X-ray computed tomography						
Image depth	bit	16	8	8	8	16	16	16
# of vol. stacks	-	7	5	3	5	5	5	7
Stack size (x)	(vx)	350	404	724	500	350	350	485
Stack size (y)	(vx)	300	385	716	500	350	350	530
Stack size (z)	(vx)	504	666	996	856	512	512	769
Voxel unit (x)	(μm)	17.86	12.74	7.02	10.0	9.2	15.04	10.2
Voxel unit (y)	(μm)	17.86	12.74	7.02	10.0	9.2	15.04	10.2
Voxel unit (z)	(μm)	17.86	12.74	7.02	10.0	9.2	15.04	10.2
Feature size (1/e $\bar{AC}$)	(vx)	1.4	1.9	2.5	2.2	1.6	1.6	1.8
Mesoscale size (10%$\bar{AC}$)	(vx)	3.4	3.0	4.3	4.2	2.7	2.7	3.3
Post-processing parameters and results								
DVC subset size (noise floor)	(vx)	17	17	25	25	17	17	17
DVC step size (noise floor)	(vx)	60	15	60	12	16	32	16
Systematic bias ($u_{x}$)	(vx)	0.009	−1.196	N/A	−0.039	−0.062	−0.064	5.722
Systematic bias ($u_{y}$)	(vx)	0.048	0.168	N/A	0.056	−0.022	−0.023	2.118
Systematic bias ($u_{z}$)	(vx)	−0.001	−0.290	N/A	−0.048	0.020	0.012	−0.234
Noise floor ($u_{x}$)	(vx)	0.013	0.029	0.023	0.021	0.022	0.023	0.038
Noise floor ($u_{y}$)	(vx)	0.012	0.030	0.017	0.020	0.020	0.023	0.032
Noise floor ($u_{z}$)	(vx)	0.013	0.027	0.031	0.019	0.019	0.020	0.029

**Table 3:** Summary of parameters for the elastomeric foam dataset under uniaxial compression [77]. Deformation was applied in steps of 7 % strain. [Dataset corresponds to Section 3.1.2]

Parameter	Unit	Landauer Foam
Material	-	Elastomeric foam
Imaging modality	-	Micro X-ray computed tomography
Image depth	bit	16
DVC feature embedding	-	Inherent pattern
Deformation	-	Steps of 7 % strain
# of vol. stacks	-	7
Stack size (x)	(vx)	987
Stack size (y)	(vx)	1009
Stack size (z)	(vx)	1856
Voxel unit (x)	(μm)	4.000
Voxel unit (y)	(μm)	4.000
Voxel unit (z)	(μm)	4.000
Feature size (1/e $\bar{AC}$)	(vx)	2.3
Mesoscale size (10 % $\bar{AC}$)	(vx)	5.0
Post-processing parameters and results		
DVC subset size (noise floor)	(vx)	65
DVC step size (noise floor)	(vx)	20
Systematic bias ($u_{x}$)	(vx)	N/A
Systematic bias ($u_{y}$)	(vx)	N/A
Systematic bias ($u_{z}$)	(vx)	N/A
Noise floor ($u_{x}$)	(vx)	0.001
Noise floor ($u_{y}$)	(vx)	<0.001
Noise floor ($u_{z}$)	(vx)	<0.001

**Table 4:** Summary of parameters for the three open-cell foam datasets. Common parameters are: Open-cell foam material, inherent pattern for DVC feature embedding, and multi-step compression. [Dataset corresponds to Section 3.1.3]

Parameter	Unit	Sriram Foam S1	Sriram Foam S2	Sriram Foam S3
Material	-	Open cell foam		
Imaging modality	-	Scanco medical micro-CT 50		
Image depth	bit	8		
DVC feature embedding	-	Inherent pattern		
Deformation	-	Multi-step compression		
# of vol. stacks	-	5	7	7
Stack size (x)	(vx)	607	571	578
Stack size (y)	(vx)	667	557	610
Stack size (z)	(vx)	600	600	600
Voxel unit (x)	(μm)		24	
Voxel unit (y)	(μm)		24	
Voxel unit (z)	(μm)		24	
Feature size (1/e $\bar{AC}$)	(vx)	14.5	13.4	15.1
Mesoscale size (10% $\bar{AC}$)	(vx)	31.0	29.6	32.9
Post-processing parameters and results				
DVC subset size (noise floor)	(vx)	151	151	151
DVC step size (noise floor)	(vx)	40	40	40
Systematic bias ($u_{x}$)	(vx)		N/A	
Systematic bias ($u_{y}$)	(vx)		N/A	
Systematic bias ($u_{z}$)	(vx)		N/A	
Noise floor ($u_{x}$)	(vx)	0.006	0.007	0.012
Noise floor ($u_{y}$)	(vx)	0.008	0.007	0.007
Noise floor ($u_{z}$)	(vx)	0.006	0.007	0.006

**Table 5:** Summary of parameters for the wood datasets. Common parameters are: Wood material, inherent patterns for DVC, and no applied deformation. [Dataset corresponds to Section 3.1.4]

Parameter	Unit	Chen Balsa	Chen Birch S1*	Chen Spruce
Material	-	Wood		
Imaging modality	-	Numerical microstructure models		
Image depth	bit	8		
DVC feature embedding	-	Inherent patterns		
Deformation	-	N/A		
# of vol. stacks	-	1		
Stack size (x)	(vx)	512	512	512
Stack size (y)	(vx)	512	512	512
Stack size (z)	(vx)	512	512	512
Voxel unit (x)	(μm)	0.24	0.51	0.48
Voxel unit (y)	(μm)	0.24	0.51	0.48
Voxel unit (z)	(μm)	0.24	0.51	0.48
Feature size (1/e $\bar{AC}$)	(vx)	20.2	9.9	11.9
Mesoscale size (10 % $\bar{AC}$)	(vx)	42.5	15.1	22.1
Post-processing parameters and results				
DVC subset size (noise floor)	(vx)	181	81	121
DVC step size (noise floor)	(vx)	30	80	60
Systematic bias ($u_{x}$)	(vx)		N/A	
Systematic bias ($u_{y}$)	(vx)		N/A	
Systematic bias ($u_{z}$)	(vx)		N/A	
Noise floor ($u_{x}$)	(vx)	0.007	0.005	<0.001
Noise floor ($u_{y}$)	(vx)	0.007	0.004	0.001
Noise floor ($u_{z}$)	(vx)	0.007	0.005	0.003

* For brevity, only the S1 variant in the dataset repository is included here.

**Table 6:** Summary of parameters for granular material datasets that were imaged using Neutron and/or XCT tomography techniques. Common parameters are: inherent patterns for DVC and deformation by rigid body motion or triaxial compression. [Dataset corresponds to Section 3.1.5]

Parameter	Unit	SPAM Sand stone	SPAM Soil	SPAM Concrete S1	SPAM Sand S2	SPAM Sand S1	SPAM Concrete S3	SPAM Concrete S1
Material	-	Sandstone	Soil	Concrete	Sand	Sand	Concrete	Concrete
Imaging modality	-	X-ray	X-ray	X-ray	Neutron	X-ray	Neutron	X-ray
Image depth	bit				16			
DVC feature embedding	-	Inherent patterns						
Deformation	-	Rigid body motion, triaxial compression						
# of vol. stacks	-	4	16	1	3	3	3	3
Stack size (x)	(vx)	551	500	250	550	550	400	400
Stack size (y)	(vx)	551	500	250	550	550	400	400
Stack size (z)	(vx)	760	800	400	610	610	375	375
Voxel unit (x)	(μm)	30	15.5	13	15.2	22.8	48.5	52.2
Voxel unit (y)	(μm)	30	15.5	13	15.2	22.8	48.5	52.2
Voxel unit (z)	(μm)	30	15.5	13	15.2	22.8	48.5	52.2
Feature size (1/e $\bar{AC}$)	(vx)	1.3	5.5	7.0	4.9	3.6	4.6	6.2
Mesoscale size (10%$\bar{AC}$)	(vx)	4.1	10.0	17.7	9.3	8.1	9.7	35.6
Post-processing parameters and results								
DVC subset size (noise floor)	(vx)	25	65	65	65	65	65	65
DVC step size (noise floor)	(vx)	12	16	64	64	64	64	64
Systematic bias ($u_{x}$)	(vx)				N/A			
Systematic bias ($u_{y}$)	(vx)				N/A			
Systematic bias ($u_{z}$)	(vx)				N/A			
Noise floor ($u_{x}$)	(vx)	0.021	0.027	0.022	0.004	0.016	0.027	0.058
Noise floor ($u_{y}$)	(vx)	0.019	0.025	0.021	0.005	0.024	0.031	0.031
Noise floor ($u_{z}$)	(vx)	0.017	0.009	0.019	0.004	0.031	0.010	0.064

**Table 7:** Summary of parameters for the two Vat photopolymer-built metamaterial datasets (“T”: transverse and “U”: upright). Common parameters are: PR-48 resin material, XCT imaging, and inherent pattern DVC. [Dataset corresponds to Section 3.1.6]

Parameter	Unit	Kafka Polymer S1 (T)	Kafka Polymer S2 (U)
Material	-	3D printed modified PR-48 resin	
Imaging modality	-	Micro X-ray computed tomography	
Image depth	bit	16	
DVC feature embedding	-	Inherent pattern	
Deformation	-	Load steps of 50 N to 100 N	
# of vol. stacks	-	8	3
Stack size (x)	(vx)	1024	
Stack size (y)	(vx)	1004	
Stack size (z)	(vx)	1016	
Voxel unit (x)	(μm)	9.00	8.25
Voxel unit (y)	(μm)	9.00	8.25
Voxel unit (z)	(μm)	9.00	8.25
Feature size (1/e $\bar{AC}$)	(vx)	9.5	4.0
Mesoscale size (10 % $\bar{AC}$)	(vx)	25.0	17.2
Post-processing parameters and results			
DVC subset size (noise floor)	(vx)	121	121
DVC step size (noise floor)	(vx)	120	120
Systematic bias ($u_{x}$)	(vx)	N/A	
Systematic bias ($u_{y}$)	(vx)	N/A	
Systematic bias ($u_{z}$)	(vx)	N/A	
Noise floor ($u_{x}$)	(vx)	0.024	0.059
Noise floor ($u_{y}$)	(vx)	0.021	0.081
Noise floor ($u_{z}$)	(vx)	0.064	0.136

**Table 8:** Summary of fabrication, post-processing, and *in situ* XCT compression testing parameters for the open-cell Kelvin lattice polymeric specimens. [Data corresponds to Section 3.1.7]

Parameter	Unit	Shaikeea Polymer
Material	-	Polymeric lattice specimens
Imaging modality	-	Micro X-ray computed tomography
Image depth	bit	8
DVC feature embedding	-	Open-cell Kelvin lattice structure
Deformation type	-	Axial Compression
# of vol. stacks	-	4
Stack size (x)	(vx)	1470
Stack size (y)	(vx)	1379
Stack size (z)	(vx)	2000
Voxel unit (x)	(μm)	40
Voxel unit (y)	(μm)	40
Voxel unit (z)	(μm)	40
Feature size (1/e $\bar{AC}$)	(vx)	0.8
Mesoscale size (10 % $\bar{AC}$)	(vx)	2.8
Post-processing parameters and results		
DVC subset size (noise floor)	(vx)	65
DVC step size (noise floor)	(vx)	64
Systematic bias ($u_{x}$)	(vx)	N/A
Systematic bias ($u_{y}$)	(vx)	N/A
Systematic bias ($u_{z}$)	(vx)	N/A
Noise floor ($u_{x}$)	(vx)	0.010
Noise floor ($u_{y}$)	(vx)	0.016
Noise floor ($u_{z}$)	(vx)	0.015

**Table 9:** Summary of parameters for the polyacrylamide hydrogel solution incorporating synthetic fluorescence beads. [Dataset corresponds to Section 3.2.1]

Parameter	Unit	Patel Beads	Yang Beads S1	Yang Beads S2	Yang Beads S3
Material	-	Synthetic data			
Imaging modality	-	Synthetic			
Image depth	bit	8			
DVC feature embedding	-	Synthetic			
Deformation	-	Sinusoidal disp.	Translation	Stretch	Rotation
# of vol. stacks	-	2	12	7	7
Stack size (x)	(vx)	512	512	512	512
Stack size (y)	(vx)	512	512	512	512
Stack size (z)	(vx)	192	192	192	192
Voxel unit (x)	(μm)	N/A	N/A	N/A	N/A
Voxel unit (y)	(μm)	N/A	N/A	N/A	N/A
Voxel unit (z)	(μm)	N/A	N/A	N/A	N/A
Feature size (1/e $\bar{AC}$)	(vx)	2.2	2.4	2.4	2.4
Mesoscale size (10 % $\bar{AC}$)	(vx)	3.4	3.9	3.9	3.9
Post-processing parameters and results					
DVC subset size (noise floor)	(vx)	41	31	31	31
DVC step size (noise floor)	(vx)	20	60	60	60
Systematic bias ($u_{x}$)	(vx)	N/A	N/A	N/A	N/A
Systematic bias ($u_{y}$)	(vx)	N/A	N/A	N/A	N/A
Systematic bias ($u_{z}$)	(vx)	N/A	N/A	N/A	N/A
Noise floor ($u_{x}$)	(vx)	0.004	0.002	0.002	0.002
Noise floor ($u_{y}$)	(vx)	0.005	0.002	0.002	0.002
Noise floor ($u_{z}$)	(vx)	0.005	0.002	0.002	0.002

**Table 10:** Summary of parameters for the polyacrylamide (PAAm) hydrogel uniaxial tension experiment. Imaging was performed with a multiphoton microscope. [Dataset corresponds to Section 3.2.2]

Parameter	Unit	Franck Polyacrylamide
Material	-	Polyacrylamide (PAAm) hydrogel
Imaging modality	-	Multiphoton microscopy
Image depth	bit	16
DVC feature embedding	-	1 μm fluorescent polystyrene particles
Deformation	-	Tension
# of vol. stacks	-	28
Stack size (x)	(vx)	1024
Stack size (y)	(vx)	1024
Stack size (z)	(vx)	126
Voxel unit (x)	(μm)	0.200
Voxel unit (y)	(μm)	0.200
Voxel unit (z)	(μm)	1.075
Feature size (1/e $\bar{AC}$)	(vx)	1.7
Mesoscale size (10 % $\bar{AC}$)	(vx)	8.0
Post-processing parameters and results		
DVC subset size (noise floor)	(vx)	31
DVC step size (noise floor)	(vx)	10
Systematic bias ($u_{x}$)	(vx)	N/A
Systematic bias ($u_{y}$)	(vx)	N/A
Systematic bias ($u_{z}$)	(vx)	N/A
Noise floor ($u_{x}$)	(vx)	0.007
Noise floor ($u_{y}$)	(vx)	0.007
Noise floor ($u_{z}$)	(vx)	0.007

**Table 11:** Summary of parameters for the indentation experiment and the synthetic datasets. The indentation was performed on a polyacrylamide hydrogel, while the other datasets are synthetic. [Dataset corresponds to Section 3.2.3]

Parameter	Unit	Yang Hydrogel
Material	-	Polyacrylamide hydrogel
Imaging modality	-	Confocal microscopy
Image depth	bit	8
DVC feature embedding	-	Fluorescence beads
Deformation type	-	Indentation
# of vol. stacks	-	2
Stack size (x)	(vx)	1024
Stack size (y)	(vx)	1024
Stack size (z)	(vx)	306
Voxel unit (x)	(μm)	0.420
Voxel unit (y)	(μm)	0.420
Voxel unit (z)	(μm)	0.425
Feature size (1/e $\bar{AC}$)	(vx)	1.9
Mesoscale size (10 % $\bar{AC}$)	(vx)	3.9
Post-processing parameters and results		
DVC subset size (noise floor)	(vx)	51
DVC step size (noise floor)	(vx)	20
Systematic bias ($u_{x}$)	(vx)	N/A
Systematic bias ($u_{y}$)	(vx)	N/A
Systematic bias ($u_{z}$)	(vx)	N/A
Noise floor ($u_{x}$)	(vx)	0.007
Noise floor ($u_{y}$)	(vx)	0.007
Noise floor ($u_{z}$)	(vx)	0.021

**Table 12:** Summary of parameters for the synthetic collagen fiber network dataset subjected to global shear. The images mimic data from a confocal microscope. [Dataset corresponds to Section 3.2.4]

Parameter	Unit	Notbohm Fibrous S2
Material	-	Synthetic fiber network image
Imaging modality	-	Synthetic (mimics confocal)
Image depth	bit	16
DVC feature embedding	-	Synthetic fluorescent fibers
Deformation	-	Global shear (5 % and 30 %)
# of vol. stacks	-	3
Stack size (x)	(vx)	383
Stack size (y)	(vx)	383
Stack size (z)	(vx)	133
Voxel unit (x)	(μm)	0.325
Voxel unit (y)	(μm)	0.325
Voxel unit (z)	(μm)	0.750
Feature size (1/e $\bar{AC}$)	(vx)	2.9
Mesoscale size (10 % $\bar{AC}$)	(vx)	29.0
Post-processing parameters and results		
DVC subset size (noise floor)	(vx)	25
DVC step size (noise floor)	(vx)	12
Systematic bias ($u_{x}$)	(vx)	N/A
Systematic bias ($u_{y}$)	(vx)	N/A
Systematic bias ($u_{z}$)	(vx)	N/A
Noise floor ($u_{x}$)	(vx)	0.008
Noise floor ($u_{y}$)	(vx)	0.007
Noise floor ($u_{z}$)	(vx)	0.005

**Table 13:** Summary of parameters for the contracting spherical inclusion experiment in a collagen fiber matrix. Imaging was performed with a spinning disk confocal microscope. [Dataset corresponds to Section 3.2.5]

Parameter	Unit	Notbohm Fibrous S1
Material	-	Collagen fibers in fluid
Imaging modality	-	Spinning disk confocal microscope
Image depth	bit	16
DVC feature embedding	-	Fluorescently-labeled fibers
Deformation	-	Contracting spherical inclusion
# of vol. stacks	-	5
Stack size (x)	(vx)	1100
Stack size (y)	(vx)	1100
Stack size (z)	(vx)	101
Voxel unit (x)	(μm)	0.325
Voxel unit (y)	(μm)	0.325
Voxel unit (z)	(μm)	0.500
Feature size (1/e $\bar{AC}$)	(vx)	7.8
Mesoscale size (10 % $\bar{AC}$)	(vx)	36.6
Post-processing parameters and results		
DVC subset size (noise floor)	(vx)	[65, 65, 17] in (x, y, z)
DVC step size (noise floor)	(vx)	32
Systematic bias ($u_{x}$)	(vx)	N/A
Systematic bias ($u_{y}$)	(vx)	N/A
Systematic bias ($u_{z}$)	(vx)	N/A
Noise floor ($u_{x}$)	(vx)	0.016
Noise floor ($u_{y}$)	(vx)	0.018
Noise floor ($u_{z}$)	(vx)	0.084

**Table 14:** Summary of parameters for human lamina cribrosa. [Dataset corresponds to Section 3.2.6]

Parameter	Unit	Nguyen Lamina*
Material	-	Posterior scleral cup and human lamina cribrosa
Imaging modality	-	Second Harmonic Generation (SHG)
Image depth	bit	8
DVC feature embedding	-	Native collagen fiber network structure
Deformation type	-	Inflation / Pressurization
# of vol. stacks	-	5
Stack size (x)	(vx)	947
Stack size (y)	(vx)	947
Stack size (z)	(vx)	52
Voxel unit (x)	(μm)	2–2.5
Voxel unit (y)	(μm)	2–2.5
Voxel unit (z)	(μm)	3
Feature size (1/e $\bar{AC}$)	(vx)	11.4
Mesoscale size (10 % $\bar{AC}$)	(vx)	24.6
Post-processing parameters and DVC results		
DVC subset size	(vx)	[41, 41, 11] in (x, y, z)
DVC step size	(vx)	10
Systematic bias ($u_{x}$)	(vx)	−0.072
Systematic bias ($u_{y}$)	(vx)	0.014
Systematic bias ($u_{z}$)	(vx)	−0.229
Noise floor ($u_{x}$)	(vx)	0.212
Noise floor ($u_{y}$)	(vx)	0.209
Noise floor ($u_{z}$)	(vx)	0.434

* For brevity, only the “Run 02: HLC_0430_LE_decon_3DCL” volume in the dataset repository is included here.

**Table 15:** Summary of parameters for the swimming animal datasets: the induced migration of *Artemia salina* imaged at two time points (S1 and S2) and a swimming *Mitrocoma* jellyfish. All datasets were acquired using Scanning Particle Image Velocimetry in seawater. [Dataset corresponds to Section 3.3.1]

Parameter	Unit	Fu Shrimp S1	Fu Shrimp S2	Fu Jellyfish
Medium	-	Seawater (38 ppt salinity)		Seawater (35 ppt salinity)
Animal	-	*Artemia salina*		*Mitrocoma cellularia*
Imaging modality	-	Scanning Particle Image Velocimetry		
Image depth	bit	16		
DVC feature embedding (Fluid)	-	13 μm silver-coated glass spheres		10 μm polyamide spheres
DVC feature embedding (Animal)	-	Inherent variations		Zinc oxide powder
Deformation	-	Fluid flow from migrating *Artemia salina* Fluid vortex from swimming *Mitrocoma*		
# of vol. stacks	-	26	26	2
Temporal Separation	(ms)	200	200	50
Stack size (x)	(vx)	1024	1024	1024
Stack size (y)	(vx)	1024	1024	1024
Stack size (z)	(vx)	757	757	491
Voxel unit (x)	(μm)	40	40	105
Voxel unit (y)	(μm)	40	40	105
Voxel unit (z)	(μm)	40	40	97
Feature size (1/e $\bar{AC}$)	(vx)	1.3	1.1	0.8
Mesoscale size (10 % $\bar{AC}$)	(vx)	3.2	2.8	1.6
Post-processing parameters and results				
DVC subset size (noise floor)	(vx)	65	65	41
DVC step size (noise floor)	(vx)	16	32	20
Systematic bias ($u_{x}$)	(vx)	N/A	N/A	N/A
Systematic bias ($u_{y}$)	(vx)	N/A	N/A	N/A
Systematic bias ($u_{z}$)	(vx)	N/A	N/A	N/A
Noise floor ($u_{x}$)	(vx)	0.012	0.017	0.045
Noise floor ($u_{y}$)	(vx)	0.014	0.020	0.045
Noise floor ($u_{z}$)	(vx)	0.109	0.123	0.157

**Table 16:** Summary of parameters for the synthetic magma compression experiment. Imaging was performed using the I12 beamline at the Diamond Light Source synchrotron. [Dataset corresponds to Section 3.3.2]

Parameter	Unit	CCPi Magma Dataset
Material	-	Synthetic Magma
Imaging modality	-	Synchrotron (Diamond Light Source, I12)
Image depth	bit	8
DVC feature embedding	-	Inherent patterns
Deformation	-	Compression
# of vol. stacks	-	2
Stack size (x)	(vx)	1520
Stack size (y)	(vx)	1257
Stack size (z)	(vx)	1260
Voxel unit (x)	(μm)	[not available]
Voxel unit (y)	(μm)	[not available]
Voxel unit (z)	(μm)	[not available]
Feature size (1/e $\bar{AC}$)	(vx)	3.6
Mesoscale size (10 % $\bar{AC}$)	(vx)	7.3
Post-processing parameters and results		
DVC subset size (noise floor)	(vx)	101
DVC step size (noise floor)	(vx)	30
Systematic bias ($u_{x}$)	(vx)	N/A
Systematic bias ($u_{y}$)	(vx)	N/A
Systematic bias ($u_{z}$)	(vx)	N/A
Noise floor ($u_{x}$)	(vx)	0.002
Noise floor ($u_{y}$)	(vx)	0.002
Noise floor ($u_{z}$)	(vx)	0.001

## Footnotes

1 Note that the current formulation removes only rigid-body translation (via the mean displacement $\mu_{j}$) and does not account for rigid-body rotation. For the static image pairs used in this study, any residual rotation between acquisitions is expected to be negligible. For datasets where rigid-body rotation may be present, a full rigid-body fit could be applied prior to computing the noise floor.
